# Effects of subsurface drip irrigation and nitrogen fertilizer management on N_2_O emissions and forage yield in alfalfa production

**DOI:** 10.3389/fpls.2025.1598110

**Published:** 2025-06-03

**Authors:** Hongxiu Ma, Quan Sun, Xiaojuan Zhang, Peng Jiang

**Affiliations:** College of Forestry and Prataculture, Ningxia University, Yinchuan, China

**Keywords:** nitrogen utilization, environmental pollution, resource utilization efficiency, crude protein, yield

## Abstract

Reducing emissions of the greenhouse gas nitrous oxide (N_2_O) while improving forage yield and quality is essential for sustainable agriculture in the context of global warming. However, how to reduce N_2_O emissions through water and nitrogen management in alfalfa planting is still unclear. In this two-year field experiment, the effects of three irrigation rates (W1, 375 mm; W2, 525 mm; W3, 675 mm) and five nitrogen (N) fertilizer application rates (N0, 0 kg N ha^−1^; N1, 75 kg N ha^−1^; N2, 150 kg N ha^−1^; N3, 225 kg N ha^−1^; N4, 300 kg N ha^−1^) on alfalfa yield, quality, resource use efficiency, and N_2_O emissions were explored. The results showed that irrigation combined with N application resulted in greater N_2_O emissions than irrigation alone. The cumulative N_2_O emissions increased with the increase of irrigation rate, and the average maximum cumulative N_2_O emissions of the W3 treatment (0.58 kg ha^−1^) increased by 94.14% and 57.38% compared with that of the W2 and W1 treatment, respectively. The cumulative N_2_O emissions also increased with the increase of the N application rate, and the average cumulative N_2_O emissions of the N4 treatment (0.69 kg ha^−1^) increased by 31.99%, 62.87%, 108%, and 173% compared with that of the N3, N2, N1, and N0 treatments, respectively. The variation of the average N_2_O emission coefficient was similar to that of the cumulative N_2_O emissions, and the W3 treatment (5.46) and N4 treatment (4.84) had the largest coefficients. Yield, crude protein, crop water productivity (WP_c_), and N_2_O emissions increased with the increase of N application rate, regardless of irrigation rate, with maxima occurring at N2 or N3 levels. These results suggest that the low NUE may be caused by the high cumulative N_2_O emissions. Besides, the combination of the irrigation rate 525 mm and the N application rate 150–225 kg N ha^-1^ could significantly increase alfalfa yield and crude protein content compared to other irrigation and nitrogen application treatments. However, further increasing irrigation and N rates failed to obtain further yield and crude protein increases, but led to N_2_O emission increase and WP_c_ and NUE reductions. This may cause serious resource waste and environmental pollution.

## 1 Introduction

Nitrous oxide (N_2_O) is a potent greenhouse gas in the atmosphere that can retain up to 121 years (Jordan et al., 2020; Wang et al., 2024). It has a global warming potential 265–298 times that of carbon dioxide (IPCC, 2014). In addition, N_2_O is a stratospheric ozone-depleting substance (Ravishankara et al., 2009). Nitrous oxide emissions from agricultural soils account for more than 60% of the global anthropogenic N_2_O emissions, and this proportion is as high as 70% in China (Tian et al., 2020). Nitrogen fertilizer is an important source of N_2_O emissions. Currently, China consumes about 30% of the world’s N fertilizer, being the world’s largest consumer, so N_2_O emissions from China’s agricultural soils cannot be ignored (Yu et al., 2019). In recent years, N fertilizer topdressing by drip fertigation has been recommended in alfalfa (*Medicago sativa*) planting. The subsurface drip fertigation can accurately supply the water and nutrients to the root zone, increase the absorption and utilization efficiency of water and nitrogen by the roots, reducing nitrogen loss (Zheng et al., 2018; Yahaya et al., 2023). However, alfalfa has a low NUE due to the great N losses caused by N_2_O emissions, ammonia volatilization, and nitrate leaching (Sehy et al., 2003; Ni et al., 2020). The large amount of N_2_O emissions not only leads to a waste of resources, but also seriously threatens the environmental security. Recent study (Tian et al., 2019) has shown that from 1860 to 2016, the global annual N_2_O emissions from chemical N fertilizers increase from 0.3 Tg N_2_O-N to 3.3 Tg N_2_O-N. Therefore, it is necessary to optimize water and N fertilizer management to reduce N_2_O emissions in alfalfa planting (Benckiser et al., 2015; Zhao et al., 2021).

Alfalfa is a legume forage widely cultivated in the arid and semi-arid regions of northwest China. Although alfalfa has a strong drought adaptability, water deficit in these regions still greatly affects its growth, dry matter yield, and quality (Lamm et al., 2012; Liu et al., 2021). Local farmers always increase alfalfa yields through over irrigation by traditional irrigation ways such as flood irrigation, resulting in high water consumption and low WP_c_ (Liu et al., 2021). This further exacerbates the water scarcity. Therefore, irrigation regime optimization is very necessary. Subsurface drip irrigation is a water-saving irrigation method. Under the premise of equal yield, subsurface drip irrigation saves 50%-60% and 20%-30% water compared with furrow irrigation and surface drip irrigation, respectively (Hassanli et al., 2008; Wang et al., 2020). This is due to that subsurface drip irrigation can directly deliver water and nutrients to plant roots and avoid surface water evaporation, thus improving the irrigation water productivity and avoiding waste of water resources (Dukes and Scholberg, 2005; Du et al., 2017). Exogenous N is a necessary for efficient and high-quality production of crops (Gao et al., 2020). In recent years, with the increase in forage demand for livestock production in China, over application of N has become a common practice for increasing alfalfa yield (Hou et al., 2021). However, over application of N reduces the positive effect, and causes greater nutrient growth than reproductive growth, thereby delaying plant maturation and reducing crop NUE and yields (Kunelius, 1974; Sun et al., 2023). This may further negatively impact the environment, ecosystem function, and biodiversity (Ren et al., 2019a; Gilles et al., 2021). Besides, soil anaerobic environment caused by over irrigation and over fertilization can accelerate the N loss by N_2_O emissions due to denitrification (Snyder et al., 2009; Li et al., 2020). Due to soil water greatly impacts crop NUE (Ren et al., 2019b), it is necessary to optimize the irrigation and N fertilization regimes to minimize the negative impacts of N loss on the environment while increasing WP_c_, NUE, and yields (Ju and Gu, 2014; Li et al., 2022).

Irrigation and N fertilization are vital for alfalfa production (Li et al., 2019). The anaerobic soil environment caused by over irrigation and the over application of N can accelerate N_2_O emissions, causing large N losses (Snyder et al., 2009; Li et al., 2020). How to optimize water and N supply to reduce N_2_O emissions while increasing alfalfa NUE, WP_c_, yield, and quality under subsurface drip irrigation remains unclear. This study hypothesized that reducing irrigation and N application rates may maximize alfalfa WP_c_, NUE, and yields while reducing the N_2_O emissions. To verify the hypothesis, this study investigated the effects of three irrigation rates and five N rates on alfalfa yield, quality, resource use efficiency, and N_2_O emissions from alfalfa fields (plants and soil) under subsurface drip irrigation. Besides, this study also clarified the optimal water and N fertilizer management in alfalfa planting. The aim was to achieve the coordination of alfalfa production and environmental protection in the arid regions of northwest China.

## 2 Materials and methods

### 2.1 Experimental site

Field experiments were conducted in 2022 and 2023 in Botanical Garden 2 Village, Liangtian Town, Yinchuan, Ningxia Hui Autonomous Region, China (106°18’ E, 38°40’ N, 1100 m a.s.l.). The experimental site has a temperate continental climate. According to the report of the Ningxia Meteorological Bureau (http://nx.cma.gov.cn/index.html), the annual sunshine duration in the experimental site was about 3032 hours, the frost-free period was 185 days, the annual average temperature was 8.7°C, the annual average precipitation was 200 mm, and the annual average evapotranspiration was 1694 mm. The physicochemical properties of surface soil (0–30 cm) sampled before the field experiment in April 2022 were determined according to the methods of Bao (2000): The soil type was aeolian sandy soil (91.76% sand, 7.04% silt, and 1.20% clay) according to the USDA soil classification. The soil pH was 8.62, the organic matter content was 4.67 g/kg, the available nitrogen content was 11.20 mg/kg, the available potassium content was 81.42 mg/kg, and the available phosphorus content was 2.44 mg/kg.

**Figure 1:**
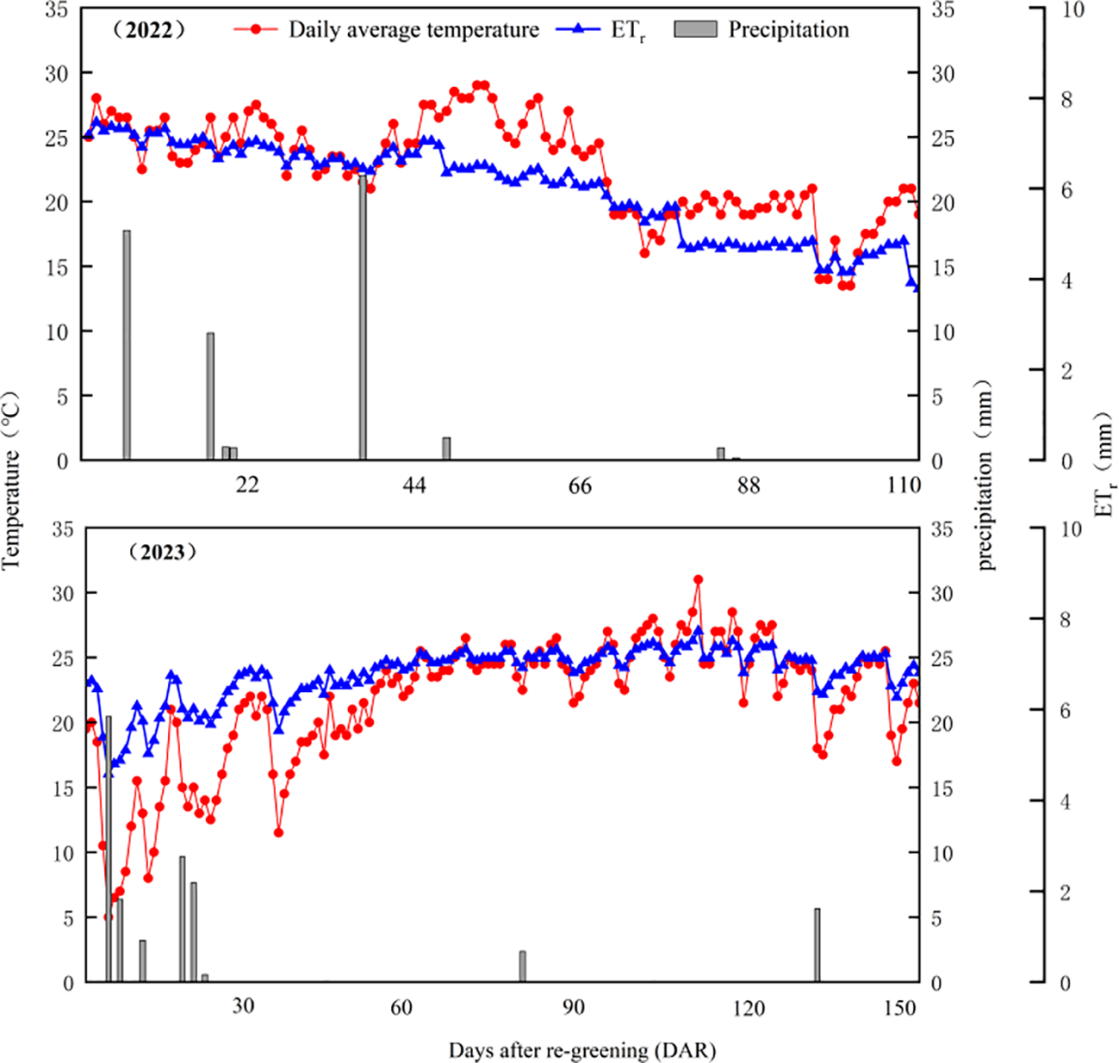
Precipitation, daily mean temperature, and reference evapotranspiration (ET_r_) during the growing seasons of alfalfa in 2022 and 2023 in the experimental site. ET_r_ is calculated according to the methods of Allen et al. (1998) and Yan et al. (2021).

Air temperature and precipitation data during both crop growing seasons were obtained from the local meteorological station (**Figure 1**). The rainfall in the growing seasons in 2022 and 2023 were 54.5 and 56.0 mm, accounting for 82.7% and 88.9% of the annual rainfall, respectively. Besides, about 50% of the rainfalls was less than 5 mm and could not be used by crops. There was no significant difference in the monthly average temperature between the two alfalfa growing seasons, with the lowest average temperature in October and the highest in July.

### 2.2 Experimental design

Alfalfa seeds (cultivar Magna Graze 401, Canada) were sown in spring in 2022, with a sowing rate of 15 kg ha^-1^ and a row spacing of 20 cm. A split-plot design was adopted, with three irrigation levels as the main plots and five N application rates as the sub-plots. The irrigation rates included 375 (W1), 525 (W2), and 675 mm (W3), and the N application rates included 0 (N0), 75 (N1), 150 (N2), 225 (N3), and 300 (N4) kg ha^-1^. There were a total of fifteen treatments, and each treatment had three replicates. The area of each plot was 12.5 m^2^ (2.5 m × 5 m). The plots were separated by vertically embedded plastic films (0–60 cm) to prevent mutual influence. The subsurface drip irrigation system used in this study was composed of a water pump, a filter, a fertilizer tank, and water pipes (inner diameter: 13 mm, wall thickness: 1.5 mm). The pipe spacing was 80 cm, the buried depth was 20 cm, the flow rate was 3.6–6 L/(m·h), and the pressure was 0.06 MPa (Xiang, 2015; Zhuge et al., 2003). Irrigation was conducted every 7 days (in case of rain or extreme heat, it was delayed or advanced by 1–2 days). A water flow meter was used to control the amount of irrigation. Alfalfa stems and leaves were harvested two times in 2022. The irrigation amount from sowing to the first harvest accounted for 60% of the total irrigation amount, and that from the first harvest to the second accounted for 40%. In the second year, alfalfa stems and leaves were harvested four times, and 25% of the total irrigation amount was irrigated before each harvest (**Supplementary Table S1**). Urea (N: 46%) was applied through the subsurface drip irrigation system after dissolving in water. The timing of N fertilization was consistent with that in local fields. Seventy percent and thirty percent of urea were applied at 2 and 67 days after emergence (Days), respectively in 2022. In the 2023, 40%, 30%, and 30% of urea were applied at 2, 45, and 73 days after leaves turning green (days), respectively. The details for irrigation and N fertilization were shown in **Supplementary Table S2**. Other agricultural managements such as weeding were the same in all plots.

### 2.3 Sampling and measurements

At the beginning of flowering (about 10% of flowering), three sampling plots (1 m × 1 m for each) were selected in the center of each plot to harvest alfalfa stems and leaves, with a stubble height of 5 cm. After that, the fresh weight was measured. The dry matter yield was measured after drying at 75°C. Hay yield was calculated on a dry matter basis (Fan et al., 2016). The dried plant samples were crushed by a pulverizer, passed through a 0.25 mm sieve, and stored in a ziplock bag at room temperature for the determination of alfalfa quality. Alfalfa N content was determined using the Kjeldahl 8400 automatic analyzer (FOSS, Hilleroed, Denmark), and the contents of neutral detergent fiber (NDF, %) and acid detergent fiber (ADF, %) were determined by the method of Raffrenato et al. (2017). Alfalfa crude protein content (CP, %) and relative feeding value (RFV) were calculated using Equations 1, 2 (Ferreira et al., 2015).


(1) CP=6.25×N

(2) RFV=(88.9−0.779×ADF)×(120/NDF)/1.29

where N is the nitrogen content of alfalfa samples (%).

### 2.4 Crop water productivity and nitrogen use efficiency

The actual crop evapotranspiration (*ET_c_*) during the growing seasons was calculated using the method of Allen et al. (1998). Due to the arid climate, flat terrain, and deep groundwater table in the experimental site, groundwater recharge, surface runoff, and deep seepage were ignored. Then, *ET*_c_ was calculated using Equation 3:


(3) $${\text{ET}}_{c}=\text{P}+\text{I}-\Delta \text{WS}$$

Where *P* is the precipitation (mm) during the alfalfa growing season, *I* is the irrigation amount (mm) during the alfalfa growing season, *ΔWS* is the change of soil water content (soil water content at the beginning of the experiment minus that at the end of the experiment (mm)).

The WP_C_ (kg m^3^) was calculated using Equation 4:


(4) $${\text{WP}}_{C}=\text{HY}/{\text{ET}}_{C}$$

The irrigation water productivity (WP_I_, kg m^3^) was calculated using Equation 5:


(5) $${\text{WP}}_{I}=\text{HY}/\text{I}$$

Where *HY* is annual hay yield (kg ha^-1^), and *I* is the total irrigation amount (mm).

Kjeldahl method (Jung et al., 2003) was used to determine the N content in alfalfa root, stems, and leaves. Plant N accumulation was calculated as the sum of N content in each organ. The agronomic efficiency of N (AEN, kg kg^-1^), N use efficiency (NUE, %), physiological efficiency of N (PEN, kg kg^-1^ N), and partial factor productivity of N (PFPN, kg kg^-1^) were calculated using Equations 6–9 (Tan et al., 2017):


(6) $$\text{AEN}=\frac{\text{Annual hay yield in N application plot}-\text{Annual hay yield in zero N plot}}{\text{N rate}}$$

(7) $$\text{NUE}=\frac{\text{Annual hay yield}}{\text{N uptake}}$$

(8) $$\text{PEN}=\frac{\text{Annual hay yield in N application plot}-\text{Annual hay yield in zero N plot}}{\text{N uptake in N application plot}-\text{N uptake in zero N plot}}$$

(9) $$\text{PFPN}=\frac{\text{Annual hay yield}}{\text{N rate}}$$

### 2.5 N_2_O collection and determination

The N_2_O fluxes from plants and soil were measured by static chamber-gas chromatography (GC) (Ning et al., 2020). The static chamber consisted of a chamber (50 cm × 50 cm × 100 cm) and a stainless steel base. Sponge and aluminum foil layers were covered on the walls of the chamber to reduce internal air temperature variations during sampling. The top of the stainless steel base was provided with a groove (2 cm in width and 5 cm in depth), which was sealed with water during gas collection. Inside the chamber was a fan and an electronic thermometer to measure the temperature of the air inside. Under normal circumstances, the soil greenhouse gas flux was measured every 7–10 days. If there was an abnormal temperature (extremely high temperature or extremely low temperature) during the alfalfa growing season, sampling frequency was increased. Besides, the timing of gas collection was postponed in case of heavy rainfall. The gas sampling was performed at 10: 00 - 14: 00 every day. Four gas samples were collected in 30 minutes (at 0, 10, 20, and 30 min) using a polypropylene syringe (50 mL) equipped with a nylon stopcock, and the samples were immediately transported to the laboratory for analysis using the Agilent gas chromatograph (7890A, USA). The N_2_O flux was calculated using Equation 10 (Kamran et al., 2022):


(10) $$\text{F}=\;\frac{M}{V0}\cdot H\cdot \frac{dc}{dt}\cdot \frac{273}{273+T}\cdot \frac{P}{P0}$$

where F is the N_2_O flux (μg N m^−2^ h^−1^), *M* is the molar mass of the measured gas (g mol^−1^), *H* is the height of the chamber (cm), *dc/dt* is the linear regression slope of gas concentration at the time approaching zero, *T* is the average temperature in the sampling chamber (°C), *P* is the pressure in the sampling chamber (Pa), and *V*0 and *P*0 are the volume (mL) and pressure (Pa) at standard conditions.

The cumulative N_2_O emissions (kg ha^−1^) was calculated using Equation 11 (Afreh et al., 2018):


(11) $$E_{c}=\sum_{i=1}^{n}\frac{F_{i+1}+F_{i}}{2}\times t_{i+1}-t_{i}\times 24$$

where *E_C_* is the cumulative N_2_O emissions during each growing season, *F* is the daily N_2_O flux, *i* is the ith measurement, (*t_i+1_-t_i_*) is the time interval between two adjacent samplings (days), and *n* is the number of observations during the growing season.

The N_2_O emission coefficient (EF, %) was calculated using Equation 12:


(12) $$\text{EF}=\frac{{\text{N}}_{2}\text{O emissions in the N application plot}-{\text{N}}_{2}\text{O emissions in the zero N plot}}{\text{N rate}}$$

### 2.6 Soil moisture and inorganic nitrogen content

To determine soil moisture and inorganic N content, three soil samples (0–20 cm) were taken with an auger near the static chamber for gas collection in each plot on the same day of gas sampling. The three soil samples were mixed and used as the sample of the plot (Wang et al., 2016). The soil moisture content was measured by weighing after drying the soil samples in an oven. The water-filled pore space (WFPS) was calculated using Equation 13 (Zhang et al., 2020):


(13) $$\text{ WFPS}\left(\%\right)=\frac{\text{soil moisture content }\%\;\times \text{soil bulk density}}{1\;-\;\frac{\text{Soil bulk density}}{2.65}}\times 100\;$$

The NH_4_^+^-N and NO_3_^–^N in the soil were extracted with 2 mol L^−1^ KCl (soil: KCl solution = 1: 5), and their contents were measured by colorimetry using a spectrophotometer (UV-2102 PCS, Shanghai Spectrometer Co., Ltd., Shanghai, China) (Wang et al., 2015).

### 2.7 Data analysis

SPSS 18.0 (IBM Corp, USA) was used for ANOVA. Tukey’s test was used to test the significance of differences in the means between treatments at *p*< 0.05 and *p*< 0.01. The direct and indirect effects of N rates and irrigation rates on N_2_O emissions, alfalfa NUE, WP_c_, yield, and quality were evaluated using a structural equation model (SEM) using the “lavaan” package in R software version 4.0.0 (R Core Team, 2020; Rosseel, 2012). The SEM was constructed based on the following assumptions: (1) Increasing the N rate might increase the N_2_O emission coefficient and the N_2_O emissions, and reduce the NUE. (2) Optimal irrigation rate and N rate could significantly improve alfalfa WP_C_, yield, and quality. The relative chi-square (χ2/df), comparative fit index (CFI), root mean square error of approximation (RMSEA), standardized root mean square residual (SRMR), Akaike information criterion (AIC), and Bayesian information criterion (BIC) were used to assess the degree of fit (Kline, 2005). Figures were drawn using Excel 2016 (Microsoft Corp, USA) and Origin 8.0 (Origin Lab Corp, USA).

## 3 Results

### 3.1 Alfalfa yield and quality

Alfalfa yield increased with the increase of irrigation rate in the two years, but there was no significant difference between W2 and W3 levels. Increasing the N rate from 0 to 225 kg N ha^-1^ resulted in a significant increase in alfalfa yield, but further increasing the N rate did not increase yield. The W2N2 treatment had the highest yield in 2022. In 2023, the W3N2 treatment had the highest yield, but there was no difference between W2N2, W2N3, W3N3, and W3N2 treatments (**Tables 1**, **2**).

**Table 1:** Effects of irrigation (W) and nitrogen (N) interaction on yield, crude protein content (CP), relative feeding value (RFV), neutral detergent fiber (NDF) content, and acid detergent fiber (ADF) content of alfalfa in 2022 and 2023.

Year	Treatment		Hay yield (t/ha)	Crude protein (%)	Relative feed value (%)	Neutral detergent fiber (%)	Acid detergent fiber (%)
2022	W1	N0	4.16 ± 0.11h	17.28 ± 0.43e	172.77 ± 8.64a	37.61 ± 1.88g	24.65 ± 1.23g
		N1	5.43 ± 0.27g	19.39 ± 0.89cd	160.67 ± 8.03b	38.68 ± 1.93fg	28.54 ± 1.43ef
		N2	6.22 ± 0.31ef	21.60 ± 1.15ab	148.98 ± 7.45bcde	40.83 ± 2.04defg	30.41 ± 1.52cde
		N3	6.66 ± 0.05de	21.15 ± 0.95ab	143.71 ± 7.19cdef	42.23 ± 2.11def	30.71 ± 1.54cde
		N4	5.23 ± 0.26g	21.68 ± 1.05ab	136.95 ± 6.85efgh	43.66 ± 2.18bcd	31.77 ± 1.59bcd
	W2	N0	5.72 ± 0.28fg	17.89 ± 0.24de	160.10 ± 8.00b	39.39 ± 1.97efg	27.17 ± 1.36fg
		N1	7.70 ± 0.27bc	21.36 ± 0.96ab	155.21 ± 7.76bc	40.15 ± 2.01defg	28.42 ± 1.42ef
		N2	8.79 ± 0.44a	22.45 ± 1.01a	145.57 ± 7.28cde	41.14 ± 2.06defg	31.59 ± 1.58bcd
		N3	7.63 ± 0.38bc	21.58 ± 0.99ab	137.37 ± 6.87efgh	42.84 ± 2.14cde	33.26 ± 1.66abc
		N4	6.95 ± 0.35d	20.60 ± 0.99bc	131.19 ± 6.56fghi	44.00 ± 2.20bcd	34.67 ± 1.73a
	W3	N0	7.02 ± 0.35d	17.81 ± 0.76de	152.25 ± 7.61bcd	41.11 ± 2.06defg	27.83 ± 1.39ef
		N1	7.77 ± 0.39b	20.81 ± 0.94abc	140.03 ± 7.00defg	43.89 ± 2.19bcd	29.42 ± 1.47def
		N2	8.08 ± 0.40b	21.47 ± 1.33ab	128.46 ± 6.42ghi	46.26 ± 2.31abc	32.40 ± 1.62abc
		N3	8.18 ± 0.42b	21.24 ± 0.63ab	124.21 ± 6.21hi	46.91 ± 2.35ab	33.83 ± 1.69ab
		N4	7.14 ± 0.27cd	17.03 ± 1.05e	119.05 ± 5.95i	48.42 ± 2.42a	34.64 ± 1.73a
Variation source							
	W		**	**	**	**	**
	N		**	**	**	**	**
	W×N		**	**	ns	ns	ns
2023	W1	N0	8.71 ± 0.62f	17.16 ± 0.53e	174.17 ± 8.71a	36.39 ± 1.82f	26.88 ± 1.34g
		N1	9.18 ± 0.55ef	18.95 ± 0.95d	164.98 ± 8.25a	37.77 ± 1.89ef	28.39 ± 1.42fg
		N2	9.80 ± 0.29bcd	20.21 ± 0.52bc	151.90 ± 7.60bc	40.03 ± 2.00cdef	30.63 ± 1.53def
		N3	9.65 ± 0.33bcde	19.68 ± 0.63bcd	146.30 ± 7.31cde	41.13 ± 2.06bcde	31.51 ± 1.58de
		N4	9.17 ± 0.21ef	19.43 ± 0.54cd	133.95 ± 6.70efg	43.75 ± 2.19abc	33.47 ± 1.67bcd
	W2	N0	9.17 ± 0.16ef	19.53 ± 0.44bcd	161.88 ± 8.09ab	38.54 ± 1.93def	28.29 ± 1.41fg
		N1	9.95 ± 0.15bc	20.62 ± 0.13bc	151.89 ± 7.59bc	40.30 ± 2.01cde	29.87 ± 1.49ef
		N2	10.87 ± 0.41a	23.30 ± 0.45a	140.95 ± 7.05cdef	42.59 ± 2.13bc	31.86 ± 1.59de
		N3	10.96 ± 0.06a	22.46 ± 0.67a	139.36 ± 6.97cdefg	42.37 ± 2.12bcd	33.01 ± 1.65cd
		N4	9.39 ± 0.32cde	20.20 ± 0.83b	129.08 ± 6.45fgh	44.67 ± 2.23ab	35.18 ± 1.76abc
	W3	N0	9.25 ± 0.19def	19.51 ± 0.32cd	147.78 ± 7.39cd	41.20 ± 2.06bcde	30.49 ± 1.52def
		N1	10.15 ± 0.32b	20.72 ± 0.83b	137.51 ± 6.88defg	43.29 ± 2.16bc	32.49 ± 1.62cde
		N2	11.00 ± 0.24a	22.76 ± 0.03a	127.89 ± 6.39fgh	45.11 ± 2.26ab	35.13 ± 1.76abc
		N3	10.88 ± 0.34a	22.66 ± 1.11a	126.21 ± 6.31gh	44.91 ± 2.25ab	36.34 ± 1.82ab
		N4	9.91 ± 0.21bc	18.52 ± 0.55d	117.73 ± 5.89h	47.57 ± 2.38a	37.27 ± 1.86a
Variation source							
	W		**	**	**	**	**
	N		**	**	**	**	**
	W×N		ns	**	ns	ns	ns

W1, W2, and W3 represent irrigation rates of 375, 525, and 675 mm, respectively, while N0, N1, N2, N3, and N4 represent nitrogen application rates of 0, 75, 150, 225, and 300 kg ha^-1^, respectively. Data are presented as mean ± SD (n = 3). Based on Tukey’s test, different lowercase letters in each column represent significant differences in means between treatments (*p*< 0.05).

**Table 2:** Effects of irrigation (W) and nitrogen (N) treatments on yield, crude protein content (CP), relative feeding value (RFV), neutral detergent fiber (NDF) content, and acid detergent fiber (ADF) content of alfalfa in 2022 and 2023.

Year	Treatment	Hay yield (t/ha)	Crude protein (%)	Relative feed value (%)	Neutral detergent fiber (%)	Acid detergent fiber (%)
2022	W1	5.52 ± 0.41b	20.22 ± 0.71a	152.62 ± 5.20a	40.60 ± 1.45b	29.22 ± 1.05b
	W2	7.47 ± 0.11a	20.77 ± 0.42a	145.89 ± 2.92a	41.50 ± 0.83b	31.02 ± 0.62ab
	W3	7.41 ± 0.34a	19.67 ± 0.68a	132.80 ± 4.74b	45.32 ± 1.57a	31.62 ± 1.09a
	N0	5.70 ± 0.24b	17.66 ± 0.35d	161.71 ± 3.23a	39.37 ± 0.79d	26.55 ± 0.53d
	N1	7.06 ± 0.13a	20.52 ± 0.41bc	151.97 ± 3.04b	40.91 ± 0.82cd	28.80 ± 0.58c
	N2	7.65 ± 0.18a	21.84 ± 0.44a	141.00 ± 2.82c	42.74 ± 0.85bc	31.46 ± 0.63b
	N3	7.50 ± 0.07a	21.32 ± 0.43ab	135.10 ± 2.70cd	43.99 ± 0.88ab	32.60 ± 0.65ab
	N4	6.03 ± 0.63b	19.77 ± 0.69c	129.06 ± 4.62d	45.36 ± 1.57a	33.69 ± 1.15a
2023	W1	9.39 ± 0.33b	19.14 ± 0.71b	154.69 ± 5.70a	39.90 ± 1.43b	30.24 ± 1.08b
	W2	10.08 ± 0.29a	21.28 ± 0.69a	145.07 ± 4.76b	41.84 ± 1.35b	31.76 ± 1.03b
	W3	10.26 ± 0.23a	20.91 ± 0.54a	131.93 ± 3.41c	44.55 ± 1.09a	34.45 ± 0.85a
	N0	9.09 ± 0.31c	18.73 ± 0.65c	161.27 ± 5.59a	38.71 ± 1.36c	28.56 ± 1.01c
	N1	9.84 ± 0.37b	20.18 ± 0.75b	152.16 ± 5.68b	40.64 ± 1.49bc	30.39 ± 1.11c
	N2	10.55 ± 0.21a	22.05 ± 0.57a	139.99 ± 3.66c	42.48 ± 1.17b	32.46 ± 0.90b
	N3	10.53 ± 0.27a	21.76 ± 0.82a	138.27 ± 5.21c	43.07 ± 1.62ab	33.83 ± 1.28ab
	N4	9.59 ± 0.35bc	19.45 ± 0.62bc	127.36 ± 4.06d	45.48 ± 1.50a	35.42 ± 1.16a

Data are presented as mean ± SD (n = 3). Based on Tukey’s test (*p*< 0.05), different lowercase letters indicate significant differences in the means between treatments. The treatment abbreviations are the same as those in **Table 1**.

The CP content increased with the increase of irrigation rate in the two years, but there was no significant difference between W2 and W3 levels. The CP content of alfalfa increased significantly from N0 to N3, but decreased significantly from N3 to N4. The average CP content of the N2 treatment was the highest, which was 24% and 18% higher than that of N0 treatment in 2022 and 2023, respectively. The W2N2 treatment had the highest CP content in 2022. In 2023, the W2N3 and W3N2 treatment had the highest CP content. The W1N0 and W3N4 treatment had the lowest CP content in both years (**Tables 1**, **2**).

The RFV value gradually decreased with the increase of irrigation rate in the two years, and the average annual RFV value was the highest at W1 level, which was 15%-17% higher than the lowest value at W3 level. The RFV value decreased with the increase of N rate. The N0 treatment had the highest RFV value, which was 25%-27% higher than the lowest value of the N4 treatment. The W1N0 treatment had the highest RFV value, and the W3N4 treatment had the lowest RFV value (**Tables 1**, **2**).

The average NDF and ADF contents were the highest at W3 level in the two years, which increased by 12% and 8%-14%, respectively compared with those at W1 treatment. With the increase of N rate, the NDF and ADF increased linearly, and the NDF and ADF contents of the N4 treatment increased by 15% - 17% and 24% - 27%, respectively compared with those of the N0 treatment in the two years. The W3N4 treatment had the highest NDF and ADF contents, and the W1N0 treatment had the lowest NDF and ADF contents (**Tables 1**, **2**).

### 3.2 Crop water productivity and nitrogen use efficiency

The *ET_C_* increased with the increase of irrigation rate in the two years, and the *ET_C_* at W3 level significantly increased by 73% and 84% in 2022 and 2023, respectively compared with that at W1 level. The WP_C_ and WP_I_ at W1 and W2 level significantly increased compared with those at W3 level. The WP_I_ and WP_C_ of the N2 and N3 treatments were the highest, and there was no difference between N2 and N3 treatments. Besides, the WP_C_ and WP_I_ of the N2 treatment increased by 13% – 33% and 15% – 38%, respectively compared with those of the N0 treatment. The W1N3 treatment had the highest WP_C_ and WP_I_ (**Tables 3**, **4**).

**Table 3:** Effects of irrigation (W) and nitrogen (N) interaction on evapotranspiration (*ET_C_*), crop water productivity (WP_C_), irrigation water productivity (WP_I_), nitrogen agronomic efficiency (AEN), nitrogen use efficiency (NUE), nitrogen physiological efficiency (PEN) and partial factor productivity of nitrogen (PFPN) of alfalfa in 2022 and 2023.

Year	Treatments		ET*_C_* (mm)	Crop Water Productivity (kg m^3^)	Irrigation water productivity (kg m^3^)	Agronomic efficiency of N (kg kg^-1^)	N use efficiency (%)	Physiological efficiency of N (kg kg^-1^)	Partial factor productivity of N (kg kg^-1^)
2022	W1	N0	201.34 ± 7.76d	20.69 ± 1.28ef	22.19 ± 0.58efg	NA	NA	NA	NA
		N1	209.44 ± 4.19cd	25.94 ± 1.81c	28.96 ± 1.45c	33.82 ± 0.68b	36.22 ± 0.72a	36.20 ± 0.72a	144.72 ± 2.89b
		N2	211.99 ± 4.24cd	29.36 ± 2.05ab	33.18 ± 1.66b	27.45 ± 0.55c	33.92 ± 0.68bc	30.05 ± 0.60b	82.91 ± 1.66e
		N3	215.23 ± 9.75c	31.00 ± 1.16a	35.56 ± 0.28a	22.56 ± 0.93d	32.66 ± 1.34cd	27.61 ± 1.13c	60.02 ± 2.47g
		N4	219.01 ± 8.81c	23.94 ± 2.15cd	27.91 ± 1.40cd	7.23 ± 0.30i	25.85 ± 1.06h	11.87 ± 0.24h	34.86 ± 0.70i
	W2	N0	282.19 ± 5.64b	21.95 ± 1.41def	23.59 ± 1.08e	NA	NA	NA	NA
		N1	289.69 ± 5.79b	26.61 ± 1.46bc	29.36 ± 1.03c	40.40 ± 0.81a	35.08 ± 0.70ab	24.66 ± 0.49d	205.42 ± 4.11a
		N2	293.27 ± 5.87b	29.99 ± 2.10a	33.49 ± 1.67b	34.64 ± 0.69b	31.27 ± 0.63def	21.15 ± 0.42e	117.15 ± 2.34c
		N3	291.19 ± 6.97b	26.22 ± 1.93c	29.07 ± 1.45c	12.95 ± 0.53g	29.82 ± 1.24ef	14.74 ± 0.61f	67.81 ± 2.82f
		N4	292.88 ± 8.01b	23.77 ± 1.83cde	26.50 ± 1.32d	5.16 ± 0.21j	30.11 ± 1.25ef	10.48 ± 0.44ij	46.42 ± 1.93h
	W3	N0	364.35 ± 8.39a	19.28 ± 1.41f	20.80 ± 1.04g	NA	NA	NA	NA
		N1	367.59 ± 7.35a	21.17 ± 1.48def	23.04 ± 1.15ef	20.22 ± 0.40g	35.23 ± 0.70ab	13.38 ± 0.27g	207.30 ± 4.15a
		N2	371.72 ± 7.43a	21.76 ± 1.52def	23.95 ± 1.20e	14.19 ± 0.28f	31.54 ± 0.63de	11.54 ± 0.23hi	107.73 ± 2.15d
		N3	374.34 ± 7.49a	21.86 ± 1.55def	24.24 ± 1.24e	10.32 ± 0.21h	29.73 ± 0.59f	10.45 ± 0.21i	72.67 ± 1.45f
		N4	375.54 ± 7.51a	19.03 ± 1.11f	21.17 ± 0.81fg	0.84 ± 0.02k	27.83 ± 0.56g	1.36 ± 0.03k	47.61 ± 0.95h
Variation source									
	W		**	**	**	**	**	**	**
	N		**	**	**	**	**	**	**
	W×N		ns	**	**	**	**	**	**
2023	W1	N0	373.73 ± 9.37c	23.29 ± 1.33c	46.47 ± 3.29c	NA	NA	NA	NA
		N1	375.19 ± 7.50c	24.49 ± 1.86abc	48.98 ± 2.91bc	12.55 ± 0.25f	60.41 ± 1.12a	12.67 ± 0.25d	122.39 ± 2.45b
		N2	380.14 ± 7.60c	25.79 ± 1.24a	52.28 ± 1.56a	14.51 ± 0.29e	52.83 ± 1.06b	15.41 ± 0.31b	65.31 ± 1.31d
		N3	384.18 ± 7.68c	25.13 ± 1.16ab	51.49 ± 1.76ab	8.47 ± 0.35g	46.82 ± 1.92c	10.14 ± 0.42e	43.46 ± 1.79f
		N4	385.92 ± 12.95c	23.78 ± 0.59bc	48.94 ± 1.12bc	3.13 ± 0.13i	45.12 ± 1.85cd	5.08 ± 0.10f	30.57 ± 0.61g
	W2	N0	533.89 ± 10.68b	17.17 ± 0.37e	34.94 ± 0.61fg	NA	NA	NA	NA
		N1	541.39 ± 10.83b	18.37 ± 0.33e	37.91 ± 0.59e	20.78 ± 0.42c	44.84 ± 0.90cd	12.68 ± 0.25d	132.61 ± 2.65a
		N2	539.89 ± 10.80b	20.13 ± 0.73d	41.42 ± 1.55d	22.68 ± 0.45b	38.37 ± 0.77fg	13.85 ± 0.28c	72.45 ± 1.45c
		N3	543.58 ± 10.77b	20.16 ± 0.29d	41.76 ± 0.23d	16.12 ± 0.66d	42.58 ± 1.75de	18.07 ± 0.74a	49.35 ± 2.03e
		N4	546.63 ± 13.19b	17.18 ± 0.53e	35.80 ± 1.22ef	1.52 ± 0.06j	40.12 ± 1.65efg	2.97 ± 0.12g	31.72 ± 1.30g
	W3	N0	700.53 ± 12.32a	13.20 ± 0.05h	27.41 ± 0.57j	NA	NA	NA	NA
		N1	710.69 ± 14.21a	14.28 ± 0.35fgh	30.08 ± 0.96hi	24.07 ± 0.48a	45.55 ± 0.91cd	14.05 ± 0.28c	135.31 ± 2.71a
		N2	710.39 ± 14.21a	15.48 ± 0.23f	32.61 ± 0.71gh	23.38 ± 0.47ab	42.58 ± 0.85de	17.99 ± 0.36a	73.33 ± 1.47c
		N3	712.94 ± 14.26a	15.26 ± 0.33fg	32.25 ± 1.00h	14.52 ± 0.29e	40.90 ± 0.82ef	15.33 ± 0.31b	48.35 ± 0.97ef
		N4	713.69 ± 14.27a	13.89 ± 0.56gh	29.38 ± 0.62ij	4.43 ± 0.09h	36.97 ± 0.74g	5.23 ± 0.10f	33.04 ± 0.66g
Variation source									
	W		**	**	**	**	**	**	**
	N		ns	**	**	**	**	**	**
	W×N		ns	ns	ns	**	**	**	*

Data are presented as mean ± SD (n = 3). Based on Tukey’s test (*p*< 0.05), different lowercase letters indicate significant differences in the means between treatments. The treatment abbreviations are the same as those in **Table 1**.

**Table 4:** Effects of irrigation (W) and nitrogen (N) treatments on evapotranspiration (ET*_C_*), crop water productivity (WP_C_), irrigation water productivity (WP_I_), nitrogen agronomic efficiency (AEN), nitrogen use efficiency (NUE), nitrogen physiological efficiency (PEN) and partial factor productivity of nitrogen (PFPN) of alfalfa in 2022 and 2023.

Year	Treatment	*ET_C_* (mm)	Crop water productivity (kg m^3^)	Irrigation water productivity (kg m^3^)	Agronomic efficiency of N (kg kg^-1^)	N use efficiency (%)	Physiological efficiency of N (kg kg^-1^)	Partial factor productivity of N (kg kg^-1^)
2022	W1	214.22 ± 8.80c	26.53 ± 1.09a	29.95 ± 1.23a	23.07 ± 0.95a	32.59 ± 1.34a	26.79 ± 1.10a	81.70 ± 3.36b
	W2	289.85 ± 5.80b	25.71 ± 0.51a	28.40 ± 0.57a	23.29 ± 0.47a	31.76 ± 0.64a	17.83 ± 0.36b	109.56 ± 2.19a
	W3	370.71 ± 7.41a	20.62 ± 0.41b	22.64 ± 0.45b	11.39 ± 0.23c	31.08 ± 0.62a	9.18 ± 0.18c	108.83 ± 2.18a
	N0	286.40 ± 11.77a	20.91 ± 0.86d	22.49 ± 0.92d	NA	NA	NA	NA
	N1	292.76 ± 12.03a	24.90 ± 1.02b	27.48 ± 1.13b	31.90 ± 1.31a	35.98 ± 1.48a	25.07 ± 1.03a	188.29 ± 7.74a
	N2	292.32 ± 5.85a	27.03 ± 0.54a	30.21 ± 0.60a	25.43 ± 0.51b	32.24 ± 0.64b	20.91 ± 0.42b	102.59 ± 2.05b
	N3	293.59 ± 5.87a	26.36 ± 0.53a	29.62 ± 0.59a	15.28 ± 0.31c	30.85 ± 0.62b	17.64 ± 0.35c	67.13 ± 1.34c
	N4	299.75 ± 12.32a	22.54 ± 0.93c	25.53 ± 1.05c	4.47 ± 0.18d	28.45 ± 1.17c	8.08 ± 0.33d	43.72 ± 1.80d
2023	W1	384.90 ± 15.81c	24.82 ± 1.02a	50.29 ± 2.07a	9.79 ± 0.40c	51.98 ± 2.14a	10.97 ± 0.45c	66.31 ± 2.72b
	W2	541.08 ± 10.82b	18.60 ± 0.37b	38.37 ± 0.77b	15.28 ± 0.31b	41.48 ± 0.83b	11.89 ± 0.24b	71.53 ± 1.43a
	W3	709.65 ± 14.19a	14.42 ± 0.29c	30.35 ± 0.61c	16.60 ± 0.33a	41.50 ± 0.83b	13.15 ± 0.26a	72.50 ± 1.45a
	N0	543.20 ± 22.32a	18.13 ± 0.74c	36.76 ± 1.51b	NA	NA	NA	NA
	N1	549.66 ± 22.58a	19.30 ± 0.79abc	39.51 ± 1.62ab	19.39 ± 0.80a	50.94 ± 2.09a	13.31 ± 0.55c	131.84 ± 5.42a
	N2	543.47 ± 10.87a	20.47 ± 0.41a	42.10 ± 0.84a	20.19 ± 0.40a	44.60 ± 0.89b	15.75 ± 0.31a	70.36 ± 1.41b
	N3	546.90 ± 10.94a	20.18 ± 0.40ab	41.83 ± 0.84a	13.04 ± 0.26b	43.43 ± 0.87bc	14.51 ± 0.29b	47.05 ± 0.94c
	N4	556.06 ± 22.85a	18.53 ± 0.76bc	38.55 ± 1.58ab	3.07 ± 0.13c	40.46 ± 0.47c	4.49 ± 0.18d	32.20 ± 1.32d

Data are presented as mean ± SD (n = 3). Based on Tukey’s test (*p*< 0.05), different lowercase letters indicate significant differences in the means between treatments. The treatment abbreviations are the same as those in **Table 1**.

In both years, the PFPN at W2 level significantly increased by 8%-34% compared with that at W1 level, but there was no difference between W3 and W1 levels. In both years, the AEN, NUE, PEN, and PFPN significantly reduced with the increase of N rate. The average AEN, NUE, PEN, and PFPN of the N4 treatment decreased by 85%, 21%, 64%, and 76%, respectively compared with those of the N0 treatment. The W3N1 treatment had the highest PFPN, and there was no difference between W3N1 and W2N1 treatment. The W1N1 treatment had the highest NUE (**Tables 3**, **4**).

### 3.3 N_2_O emissions, soil moisture content, and inorganic nitrogen content

**Figure 2:**
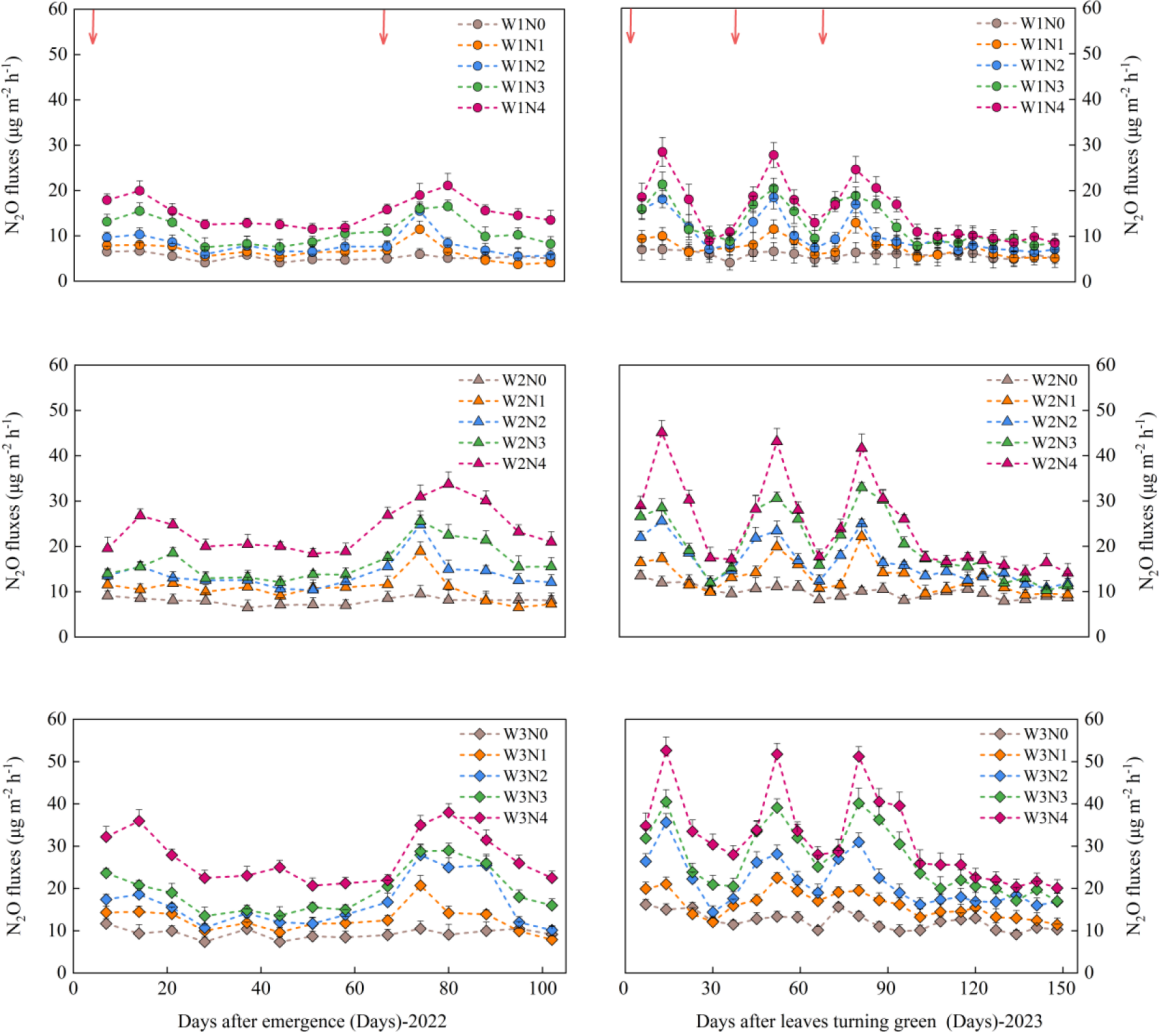
Effects of irrigation (W) and nitrogen (N) treatments on N_2_O fluxes during the alfalfa growing seasons in 2022 and 2023. Error bars represent standard deviation (SD). The red arrows indicate fertilization events. W1, W2, and W3 represent irrigation rates of 375, 525, and 675 mm, respectively, and N0, N1, N2, N3, and N4 represent nitrogen application rates of 0, 75, 150, 225, and 300 kg ha^-1^, respectively.

In 2022, N_2_O flux peaks were observed at 14 and 74 days (12 and 7 days after N topdressing, respectively). In 2023, N_2_O flux peaks were observed at 14, 52, and 80 days (12, 7, and 7 days after the first, second, and third N topdressing). In the later stages of crop growth, irrigation and N treatments had little effect on N_2_O flux (**Figure 2**).

**Figure 3:**
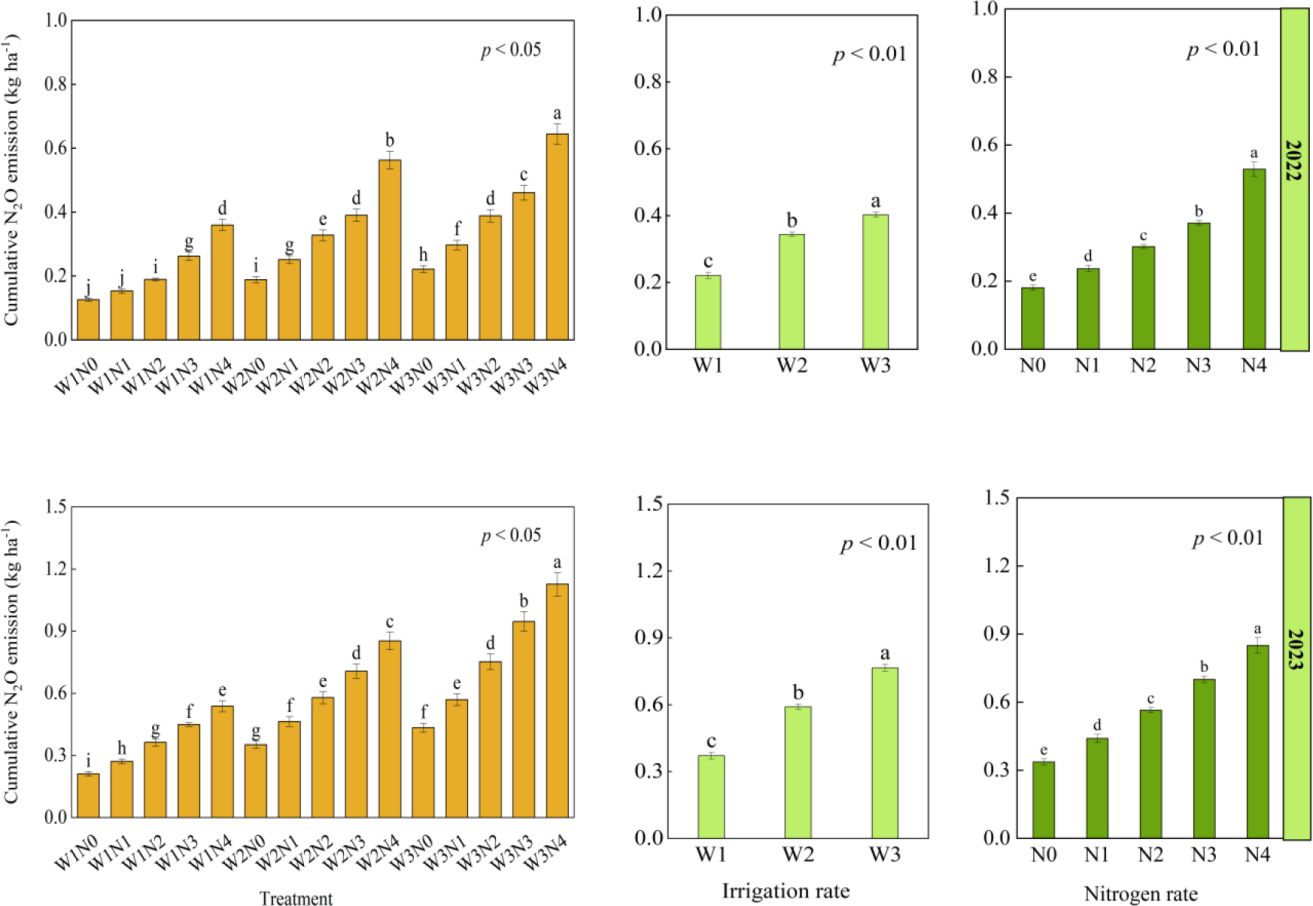
Effects of irrigation (W) and nitrogen (N) treatments on the cumulative N_2_O emissions during the alfalfa growing seasons in 2022 and 2023. Different lowercase letters indicate significant differences between treatments at *p*< 0.05 (Tukey’s test). The treatment abbreviations are the same as those in **Figure 2**.

Irrigation (W), N fertilization (N), and their interaction (W × N) had significant effects on the cumulative N_2_O emissions. With the increase of irrigation and N rates, the cumulative N_2_O emissions showed an increasing trend. In 2022 and 2023, the cumulative N_2_O emissions at W3 level increased by 82% and 106%, respectively compared with that at W1 level, and the cumulative N_2_O emissions of the N4 treatment increased by 192% and 153%, respectively compared with that of the N0 treatment (**Figure 3**). The W3N4 treatment had the highest cumulative N_2_O emissions, and the W1N0 treatment had the lowest cumulative N_2_O emissions (**Figure 3**). The change of N_2_O emission coefficient was similar to that of the cumulative N_2_O emissions in the two years. The W3N4 treatment had the highest N_2_O emission coefficient.

In the two years, irrigation and N fertilization had no significant effect on WFPS values due to the short irrigation interval. However, the WFPS value increased with the increase of irrigation rate, and the WFPS value of the N1 treatment decreased compared with that of the N0 treatment at each irrigation level (**Supplementary Figure S1**).

Soil inorganic N content showed similar dynamics in the two years. In 2022 and 2023, the content of NH_4_^+^-N ranged from 2.2 to 4.0 mg kg^-1^ and the content of NO_3_^–^N ranged from 2.5 to 7.5 mg kg^-1^ in the surface soil (**Supplementary Figure S2**, **S3**). Nitrogen application increased soil inorganic N contents compared with N0 treatment. Peaks in NO_3_^–^N content was observed during 10–17 and 71–77 days for all treatments in the first year, and during 10-17, 49-55, and 77–83 days in the second year (**Supplementary Figure S3**). The NO_3_^–^N content was maintained at a high level under W3N4 treatment, and the irrigation treatment alone had no significant effect on the contents of NH_4_^+^-N and NO_3_^–^N. soil N_2_O flux was significantly positively correlated with WFPS, NH_4_^+^-N content, and NO_3_^–^N content during the two growing seasons (**Supplementary Figure S4**).

### 3.4 Pearson correlation analysis and structural equation modeling results

**Figure 4:**
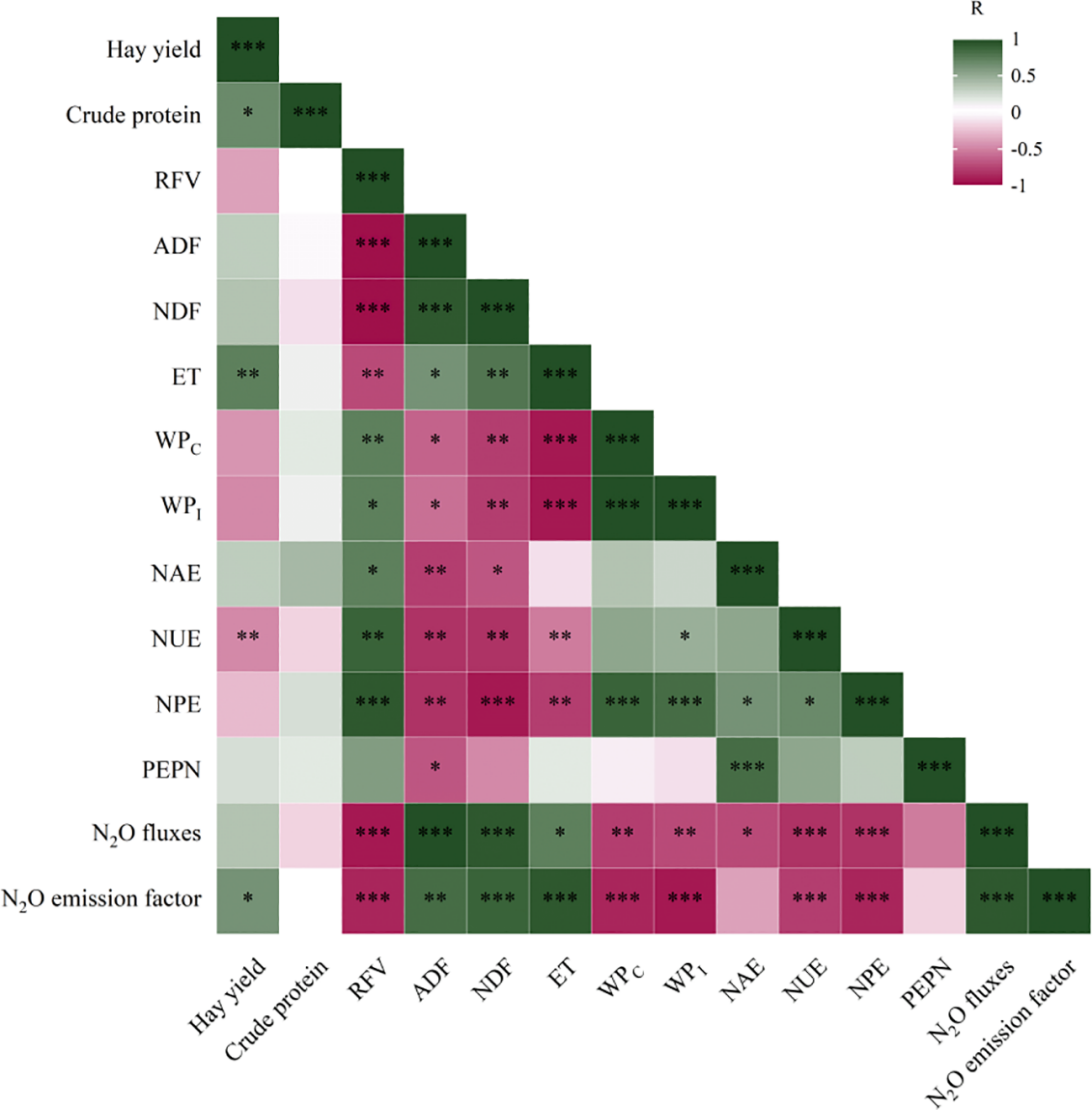
Correlation analysis of alfalfa yield, crude protein (CP), relative feeding value (RFV), acid detergent fiber (ADF), neutral detergent fiber (NDF), evapotranspiration (*ET_C_*), crop water productivity (WP_C_), irrigation water productivity (WP_I_), nitrogen agronomic efficiency (AEN), nitrogen use efficiency (NUE), nitrogen physiological efficiency (PEN), partial factor productivity of nitrogen (PFPN), N_2_O flux, and N_2_O emission coefficient. Red and blue represent negative and positive correlations, respectively. *p< 0.05; **p< 0.01; ***p< 0.001.

Most of the NUE indicators including AEN, NUE, PEN, and PFPN were positively correlated with RFV, WP_C_, and WP_I_, and negatively correlated with ADF, RDF, N_2_O flux, and N_2_O emission coefficient (**Figure 4**). Alfalfa yield was significantly positively correlated with CP, *ET_C_*, and N_2_O emission coefficient.

**Figure 5:**
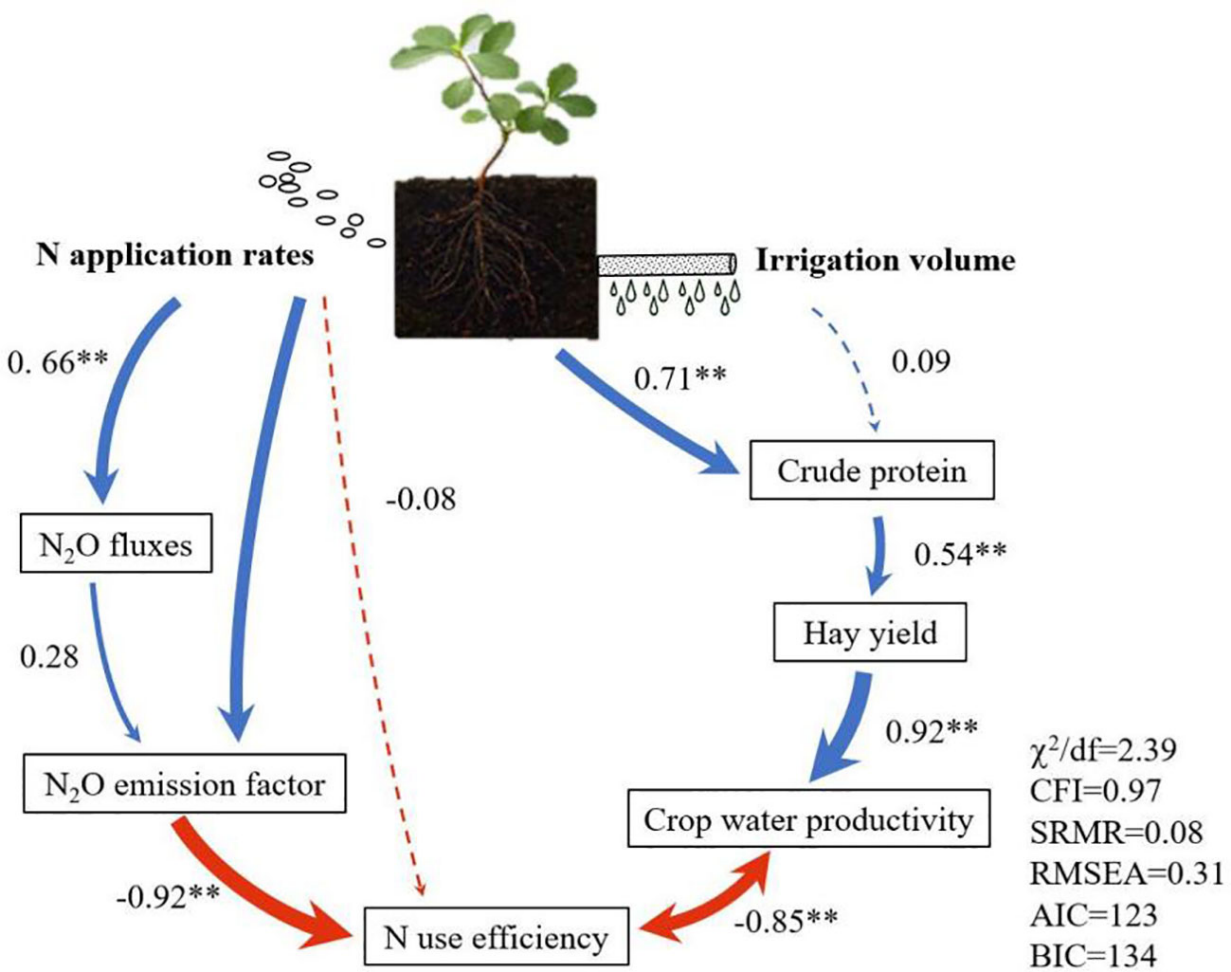
Structural equation modeling (SEM) for the effects of irrigation rate and nitrogen application rate on N_2_O emissions, N_2_O emission coefficients, nitrogen use efficiency, crop water productivity, yield, and crude protein content. The numbers adjacent to the arrows are the factor loading, which explains the variance of the observed variable, and the width of the line is proportional to the factor loading. The red and blue lines indicate negative and positive effects, respectively. Critical paths are marked with *.

The SEM model showed that increasing N rate resulted in an increase in N_2_O emissions and a decrease in NUE, with factor loading of 0.66 and -0.92, respectively (*p*< 0.01). In addition, irrigation and N fertilization significantly increased CP content, yield, and WP_C_, with factor loading of 0.71, 0.54, and 0.92, respectively (*p*< 0.01). Overall, the results supported the hypothesis of the model, that is moderately reducing irrigation and N application rates may maximize water and nutrient use efficiency and alfalfa yields while reducing the N_2_O emissions. (**Figure 5**).

## 4 Discussion

Nitrous oxide emissions from farmland are affected by multiple factors, such as climatic factors, soil properties, and agricultural managements (Akiyama et al., 2009; Cai and Akiyama, 2017). The results of this study showed that there were different N_2_O flux peaks in each growing season. This is directly related to the increase of soil NO_3_^−^ (2.5-7.5 mg N kg^-1^) after N fertilization. Interestingly, the first peak N_2_O flux occurred 12 days after the first N topdressing in the two years, and another peaks occurred 7 days after the second and third N topdressing. This may be related to the temperature change during the growing season. The increase in temperature during the second and third N topdressing can enhance the respiration of microorganisms, causing soil oxygen deficit. Denitrifying microorganisms utilize nitrate in soil as an electron acceptor and reduce it to nitrogen through a series of enzymatic reactions, accelerating the denitrification (Braker et al., 2010; ChengHsien et al., 2020). Therefore, the peaking time of N_2_O flux is significantly earlier than that after N application in spring. In addition, after N topdressing, the sufficient soil inorganic N, particularly nitrate nitrogen, provides more substrates for denitrifying microorganisms, accelerating the denitrification. This may also be an important reason for the early appearance of peak N_2_O flux (Millar et al., 2018; Schellenberg et al., 2012). It was also found that after the fourth harvest at 106 days in the second year, no significant N_2_O fluxes were observed after irrigation alone. This may be due to the fact that the substrates such as organic carbon and nitrogen in the soil are diluted or lost, resulting in insufficient substrates for microorganisms. This limits the growth and metabolism of microorganisms, and reduces their activities (Li et al., 2020). In this study, irrigation combined with N fertilization caused higher N_2_O emissions than irrigation alone. This may be due to the fact that high soil moisture content hinders gas diffusion, and causes an anaerobic soil environment. This makes the metabolic activities of denitrifying microorganisms more active, and increases soil denitrification potential and rate, i.e., reducing nitrate nitrogen to gaseous nitrogen more quickly, thus increasing N_2_O emissions (Sainju et al., 2012). In this study, compared with high irrigation rate W3 (675 mm), the irrigation rate 375–525 mm was more conducive to improving soil permeability and microbial environment, thereby inhibiting denitrification and reducing N_2_O emissions (Abalos et al., 2014). High N application rates resulted in higher accumulative N_2_O emissions and higher N_2_O emission coefficients than other nitrogen application rate treatments in this study. This may be due to the fact that most of the applied N could not be absorbed and utilized by alfalfa, and the N residues in soil are used by soil microorganisms for nitrification and denitrification (Liu et al., 2017; Lyu et al., 2019), thereby increasing N_2_O emissions. It was found that when the N application rate was increased to 300 kg N ha^-1^, the two-year average N_2_O emission coefficient increased to 5% compared with that of the N1 treatment. Therefore, reducing the N application rate is an effective way to reduce the N_2_O emission in alfalfa planting, and N2 may be the optimal N rate because the two-year average N_2_O emissions of the N2 treatment could be significantly reduced by 63% compared with that of the N4 treatment.

Soil inorganic N is the main source of microbial N_2_O production (Millar et al., 2018; Xiao et al., 2018). This study results showed that the N_2_O flux peak was significantly enhanced after irrigation combined with N fertilization, and the soil NO_3_^–^N content was high during N_2_O flux peaks, and the high soil NO_3_^–^N content lasted for about two weeks after N topdressing. This result was validated by the correlation analysis results, that is, there was a significant positive correlation between N_2_O fluxes and NO_3_^–^N (R^2^ = 0.82)/NH_4_^+^-N (R^2^ = 0.21) content.

Optimizing WP_C_ is one of the focus of this study. It can be achieved by reducing *ET_C_* and increasing alfalfa yield (Li et al., 2019). In this study, the WP_C_ at W3 level was lower than that at W1 and W2 levels. This may be due to the increased soil *ET_C_* and percolation (**Table 4**) (Li et al., 2019; Cai et al., 2020). Besides, it was found that the effect of N application rates on *ET_C_* was not significant, but the N application rate of 150–225 kg N ha^-1^ significantly increased alfalfa yield, so both WP_C_ and WP_I_ can be maximized. In 2023, at different irrigation levels, NAE increased significantly with the increase of irrigation rate, while NUE showed a downward trend. This may be due to the fact that under drought conditions, irrigation promotes alfalfa growth, and NAE continues to rise due to the release of yield potential. However, after exceeding the optimal irrigation rate, NUE decreases due to nitrogen losses through leaching and denitrification. This contradiction highlights the importance of water-nitrogen coupling optimization in alfalfa planting. The PFPN, AEN, NUE, and NP of the N4 treatment decreased compared with those of the N1 treatment. This is mainly due to that the imbalance between alfalfa N requirement and N supply (Liu et al., 2015) inhibits the growth and development of alfalfa roots, reduces the uptake of nutrients and water, and ultimately affects alfalfa yield (Islam et al., 2012). Therefore, the N application rate of 75–225 kg ha^-1^ is more conducive to promoting root development and root activity, regulate the distribution of photo assimilates in plant shoots, and effectively improve alfalfa resource use efficiency and yield, compared with other N application rates (Vasileva and Pachev, 2015). Mumford et al. (2019) reported that N_2_O emissions from dry farmlands are an important pathway for N loss and the main cause of low NUE. This is confirmed by the negative correlation between N_2_O emissions and NUE in this study (**Figure 4**). In conclusion, both over irrigation and over N application could affect alfalfa WP_C_ and NUE, and the optimal irrigation(W2, 525 mm) and N application rates(N2/N3, 150–225 kg ha^-1^) could achieve high resource use efficiency.

In arid and semi-arid regions, irrigation and fertilization are the main determinants of forage yield and quality (Djaman et al., 2020). The results of this study showed that the rainfalls during the growing season of alfalfa in 2022 (54.5 mm) and 2023 (56.0 mm) were low, and increasing the irrigation rate significantly increased alfalfa yield. This is due to that sufficient water and nutrient supply improves alfalfa leaf photosynthesis, thus increasing alfalfa biomass (Ferreira et al., 2015; Li and Su, 2017). However, the subsurface drip irrigation can reduce water evaporation, so the irrigation rate W2 is sufficient to meet the water needs of crop growth, and further increasing the irrigation rate has no significant effect on alfalfa yield.

The nutritional quality of forage determines the value in use and value in exchange, because it affects the digestion of forage, the energy and nutrient absorption by livestock, and ultimately the yield and quality of livestock products (Richman et al., 2015). Crude protein content (CP), relative feed value (RFV), neutral detergent fiber (NDF), and acidic detergent fiber (ADF) are important indicators to measure the nutritional quality of forage (McDonald et al., 2021). In this study, the change trend of CP content with irrigation rate was similar to that of yield, while the contents of NDF and ADF increased significantly at W3 level compared with those at W1 and W2 levels. This may be due to that over irrigation accelerates crop maturation, reduces CP content, and increases cell wall contents and fiber count (Liu et al., 2021). Compared with the N0 treatment, applying 150–225 kg ha^-1^ of N fertilizer significantly improved alfalfa yield, CP content, and RFV. However, further increasing N application rate led to a decrease in alfalfa yield, CP content, and RFV. This may be due to that the soil available N content is low (11.2 mg kg^−1^) at the experimental site. N application can increase the chlorophyll content and photosynthetic capacity of leaves, which increases the dry matter yield and the synthesis of amino acids, thus improving the protein content of alfalfa (Gao et al., 2020). However, excessive N inputs can affect nodulation and N fixation, but can also be counterproductive to crop growth (Xie et al., 2015; Reinprecht et al., 2020).

According to recent survey, most farmers in the experimental site applied 450 kg ha^-1^ of N to pursue high yield. This adversely affects alfalfa quality, resource utilization, and environmental health (Fan et al., 2016; Sha et al., 2021). When assessing the feasibility of agricultural managements such as irrigation and N fertilization, it is important to consider not only their impacts on crop yields, but also their impacts on the environment (Tan et al., 2017; Zhang et al., 2022). In general, the irrigation rate of 525 mm combined with the N application rate of 150–225 kg N ha^-1^ could increase alfalfa yield, quality, and resource use efficiency, while reducing N_2_O emissions. Thus, it is the optimal combination for local alfalfa planting under subsurface drip irrigation.

## 5 Conclusion

The cumulative N_2_O emissions showed an increasing trend with the increase of irrigation and N application rates. High cumulative N_2_O emissions are an important reason for the low NUE. The irrigation rate of 525 mm and the N application rate of 150–225 kg ha^-1^ could significantly improve the yield and quality of alfalfa compared with the over irrigation(W3, 675 mm) and over N fertilization(N4, 300 kg ha^-1^) by local farmers. However, further increasing the irrigation and N application rates could not further increase the yield and quality of alfalfa, but caused an increase in N_2_O emissions and a decrease in WP_C_ and NUE. This may cause serious resource waste and environmental pollution. However, rainfall and soil texture are different in different arid regions. This may significantly affect the relationship between resource use and greenhouse gas emissions during the growing season of alfalfa. Therefore, it is necessary to clarify the response of resource use efficiency to climate change under different precipitations and soil types in the future, to further optimize irrigation and fertilization strategies.

## Data Availability

The original contributions presented in the study are included in the article/**Supplementary Material**. Further inquiries can be directed to the corresponding author.
