# Supplementary material for: Effects of subsurface drip irrigation and nitrogen fertilizer management on N2O emissions and forage yield in alfalfa production
Source: Front Plant Sci. 2025 Jun 3;16:1598110. doi: 10.3389/fpls.2025.1598110 (PMC12172550; doi:10.3389/fpls.2025.1598110)

**Table S1.** Harvesting, irrigation, and nitrogen application schedule for the alfalfa growing season (2022-2023).

| **Year** | **Harves** | **Days after re-greening**  **(Days)** | **Date** | **Irrigation** | **Nitrogen application** |
| --- | --- | --- | --- | --- | --- |
|  |  |  |  |  |  |
| **2022** | 1st | 0-64 | 10 May -12 July | 60% | 70% |
|  | 2nd | 65-108 | 13 July - 25 August | 40% | 30% |
| **2023** | 1st | 0-39 | 17 April-25 May | 25% | 40% |
|  | 2nd | 40-70 | 26 May-25 June | 25% | 30% |
|  | 3rd | 71-105 | 26 June-30 July | 25% | 30% |
|  | 4th | 106-145 | 31 July-8 September | 25% | - |

***** The values refer to the percentage of the total amount for different irrigation and N amounts.

**Table S2.** Irrigation and nitrogen management strategy during the two growing seasons.

| Year/Date (M-D) | | Days after re-greening | Irrigation amount（mm） | | |  | Nitrogen fertilizer amount (kg/ha) | | | | |
| --- | --- | --- | --- | --- | --- | --- | --- | --- | --- | --- | --- |
|  |  |  | W1 | W2 | W3 |  | N0 | N1 | N2 | N3 | N4 |
| 2022 | 05-10 | 0 | 45 | 45 | 45 |  | - | - | - | - | - |
|  | 05-12 | 2 | 20 | 30 | 40 |  | - | 52.5 | 105 | 157.5 | 210 |
|  | 05-18 | 8 | 20 | 30 | 40 |  | - | - | - | - | - |
|  | 05-25 | 15 | 20 | 30 | 40 |  | - | - | - | - | - |
|  | 06-02 | 22 | 20 | 30 | 40 |  | - | - | - | - | - |
|  | 06-09 | 29 | 20 | 30 | 40 |  | - | - | - | - | - |
|  | 06-16 | 36 | 20 | 30 | 40 |  | - | - | - | - | - |
|  | 06-23 | 43 | 20 | 30 | 40 |  | - | - | - | - | - |
|  | 06-30 | 50 | 20 | 30 | 40 |  | - | - | - | - | - |
|  | 07-08 | 58 | 20 | 30 | 40 |  | - | - | - | - | - |
|  | 07-17 | 67 | 25 | 35 | 45 |  | - | 17.5 | 45 | 67.5 | 90 |
|  | 07-23 | 73 | 25 | 35 | 45 |  | - | - | - | - | - |
|  | 07-29 | 79 | 25 | 35 | 45 |  | - | - | - | - | - |
|  | 08-06 | 87 | 25 | 35 | 45 |  | - | - | - | - | - |
|  | 08-13 | 94 | 25 | 35 | 45 |  | - | - | - | - | - |
|  | 08-20 | 101 | 25 | 35 | 45 |  | - | - | - | - | - |
|  | Total |  | 375 | 525 | 675 |  | - | - | - | - | - |
| 2023 | 04-19 | 2 | 35 | 35 | 35 |  | - | 30 | 60 | 90 | 120 |
|  | 04-24 | 7 | 17 | 24.5 | 32 |  | - | - | - | - | - |
|  | 05-01 | 14 | 17 | 24.5 | 32 |  | - | - | - | - | - |
|  | 05-10 | 23 | 17 | 24.5 | 32 |  | - | - | - | - | - |
|  | 05-17 | 30 | 17 | 24.5 | 32 |  | - | - | - | - | - |
|  | 05-24 | 37 | 17 | 24.5 | 32 |  | - | - | - | - | - |
|  | 06-01 | 45 | 17 | 24.5 | 32 |  | - | 22.5 | 45 | 67.5 | 90 |
|  | 06-08 | 52 | 17 | 24.5 | 32 |  | - | - | - | - | - |
|  | 06-15 | 59 | 17 | 24.5 | 32 |  | - | - | - | - | - |
|  | 06-22 | 66 | 17 | 24.5 | 32 |  | - | - | - | - | - |
|  | 06-29 | 73 | 17 | 24.5 | 32 |  | - | 22.5 | 45 | 67.5 | 90 |
|  | 07-06 | 80 | 17 | 24.5 | 32 |  | - | - | - | - | - |
|  | 07-13 | 87 | 17 | 24.5 | 32 |  | - | - | - | - | - |
|  | 07-20 | 94 | 17 | 24.5 | 32 |  | - | - | - | - | - |
|  | 07-27 | 101 | 17 | 24.5 | 32 |  | - | - | - | - | - |
|  | 08-03 | 108 | 17 | 24.5 | 32 |  | - | - | - | - | - |
|  | 08-10 | 115 | 17 | 24.5 | 32 |  | - | - | - | - | - |
|  | 08-15 | 120 | 17 | 24.5 | 32 |  | - | - | - | - | - |
|  | 08-22 | 127 | 17 | 24.5 | 32 |  | - | - | - | - | - |
|  | 08-29 | 134 | 17 | 24.5 | 32 |  | - | - | - | - | - |
|  | 09-05 | 141 | 17 | 24.5 | 32 |  | - | - | - | - | - |
|  | Total |  | 375 | 525 | 675 |  | - | - | - | - | - |

W1, W2, and W3 represent irrigation rates of 375, 525, and 675 mm, and N0, N1, N2, and N3 represent nitrogen application rates of 0, 75, 150, 225, and 300 kg ha^–1^, respectively. Data are presented as means ± SD (n = 4). Different lowercase letters within each column represent significant differences in the means between treatments based on Tukey’s test (*p* ≤ 0.05).

**Figure S1.** Effects of irrigation (W) and nitrogen (N) treatments on the dynamics of average soil water-filled pore space (WFPS) during alfalfa growing seasons in 2022 and 2023. Error bars indicate standard deviation (SD). The red arrow indicates a fertilization event. The processing abbreviations are the same as those described in Figure 2.


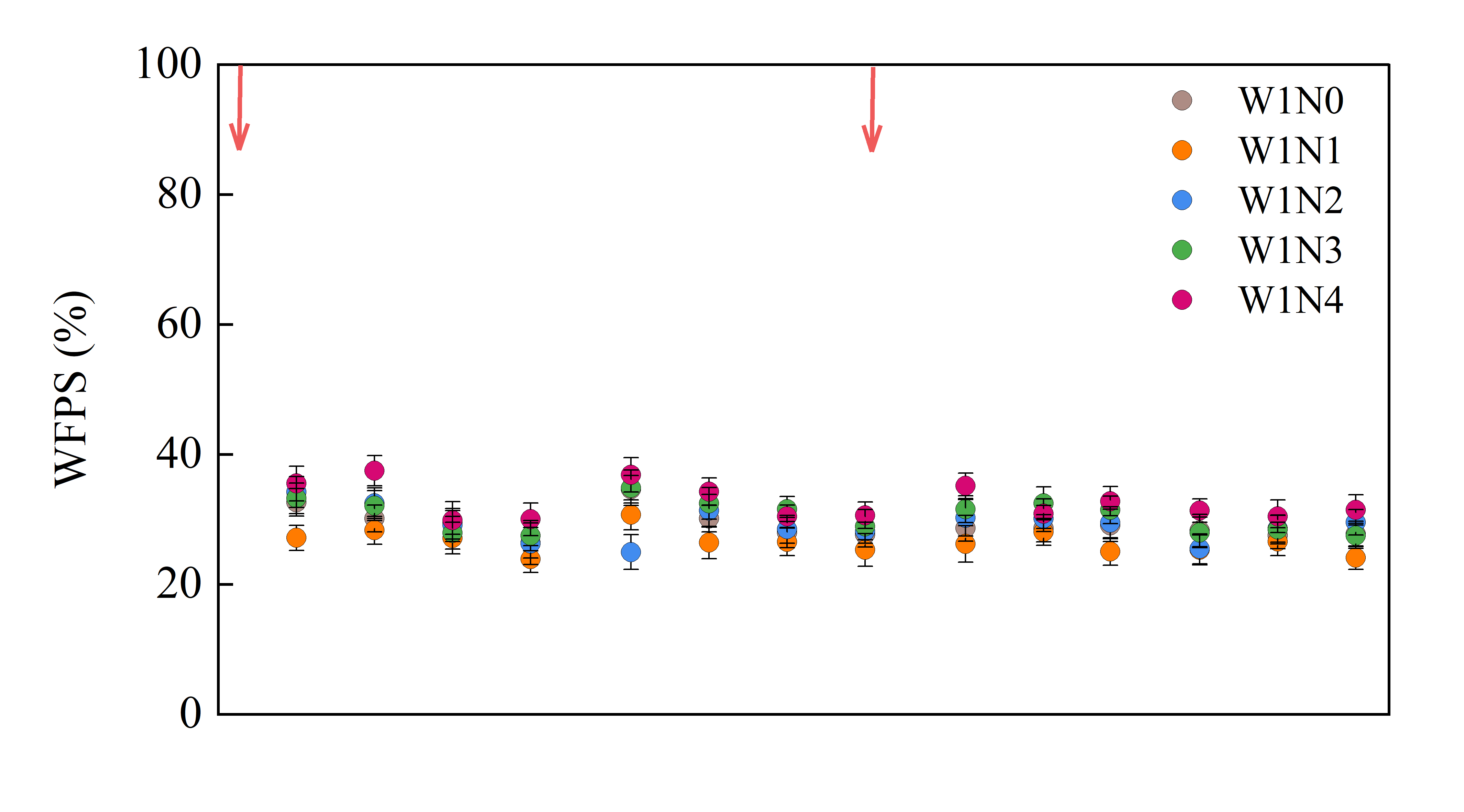

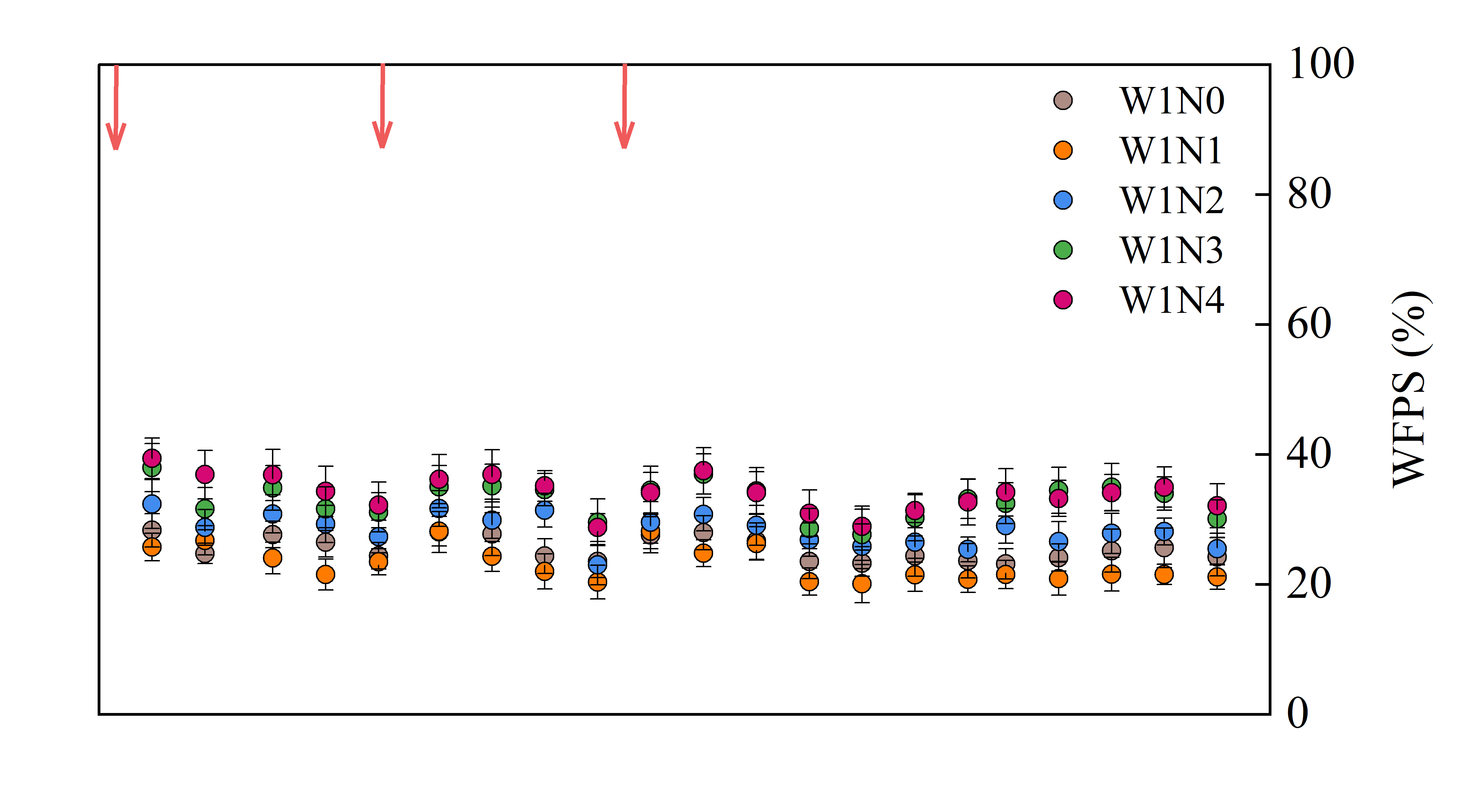

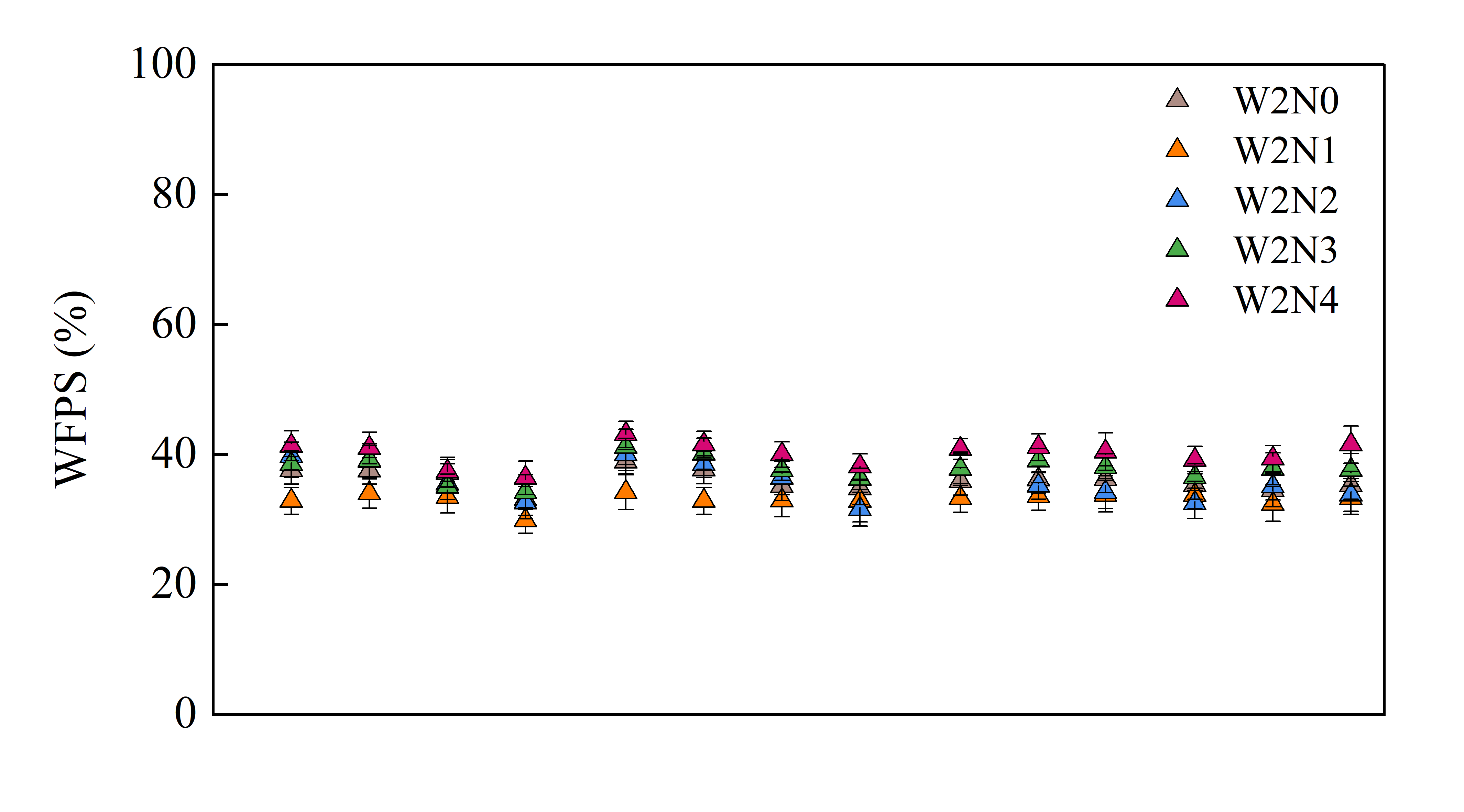

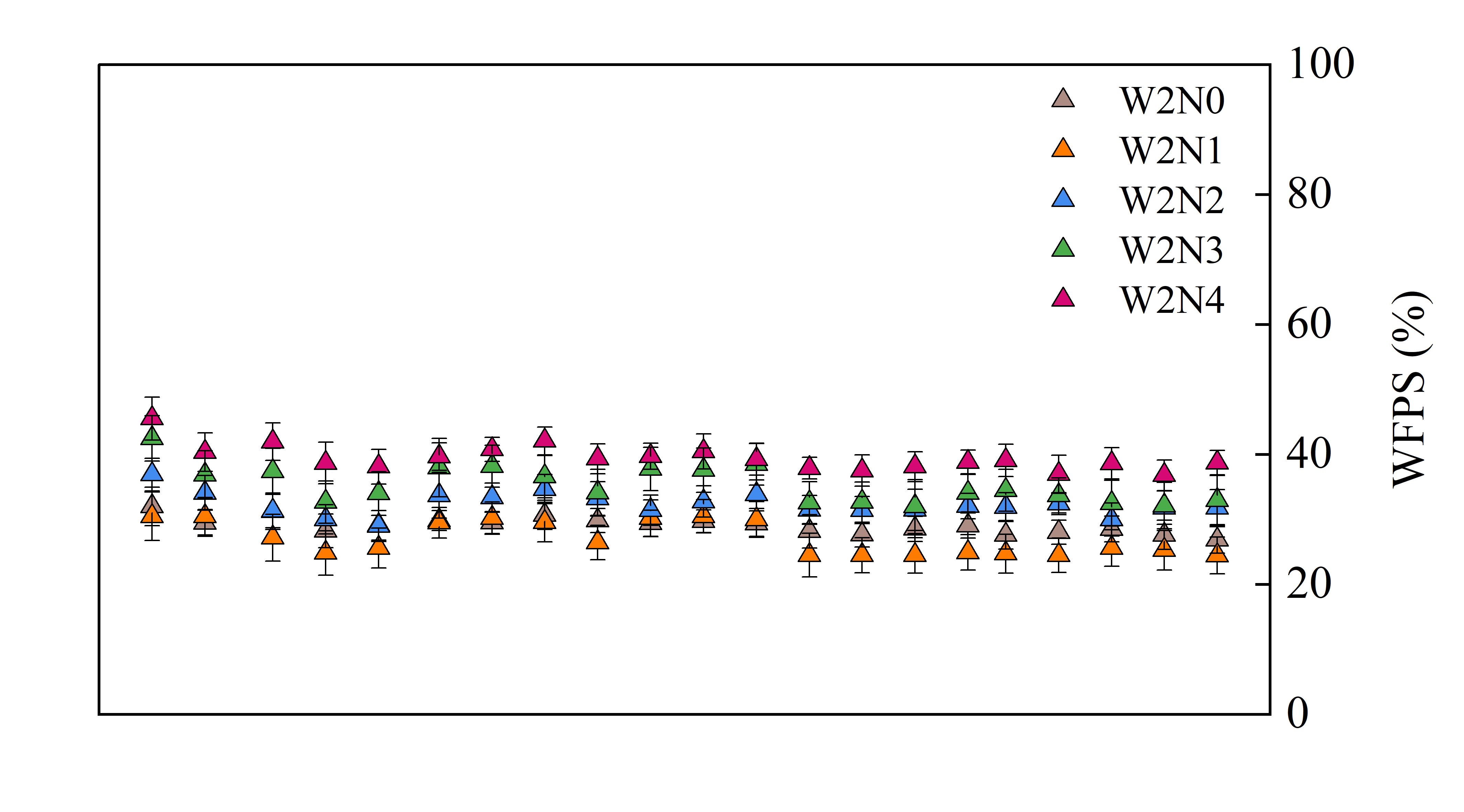

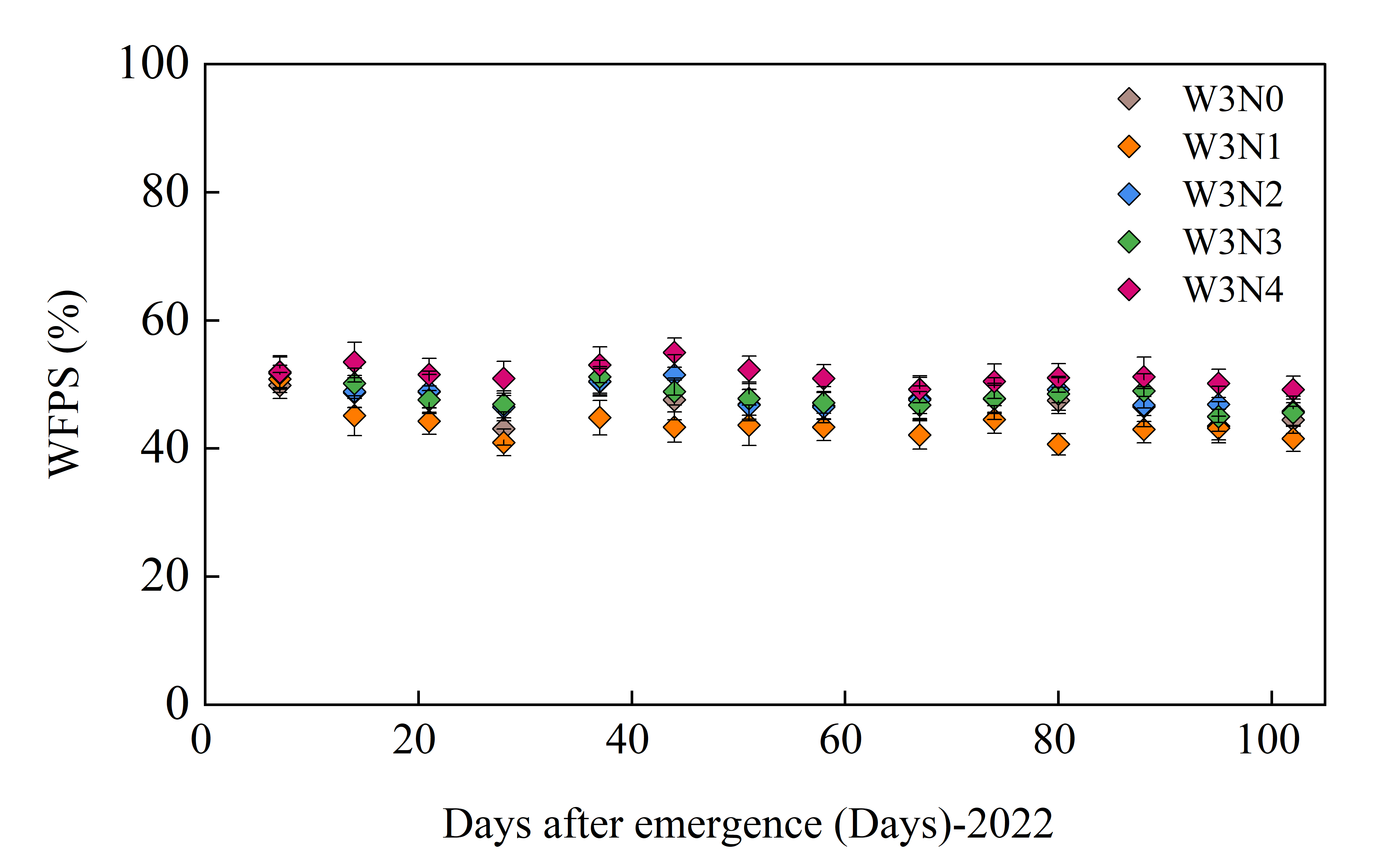

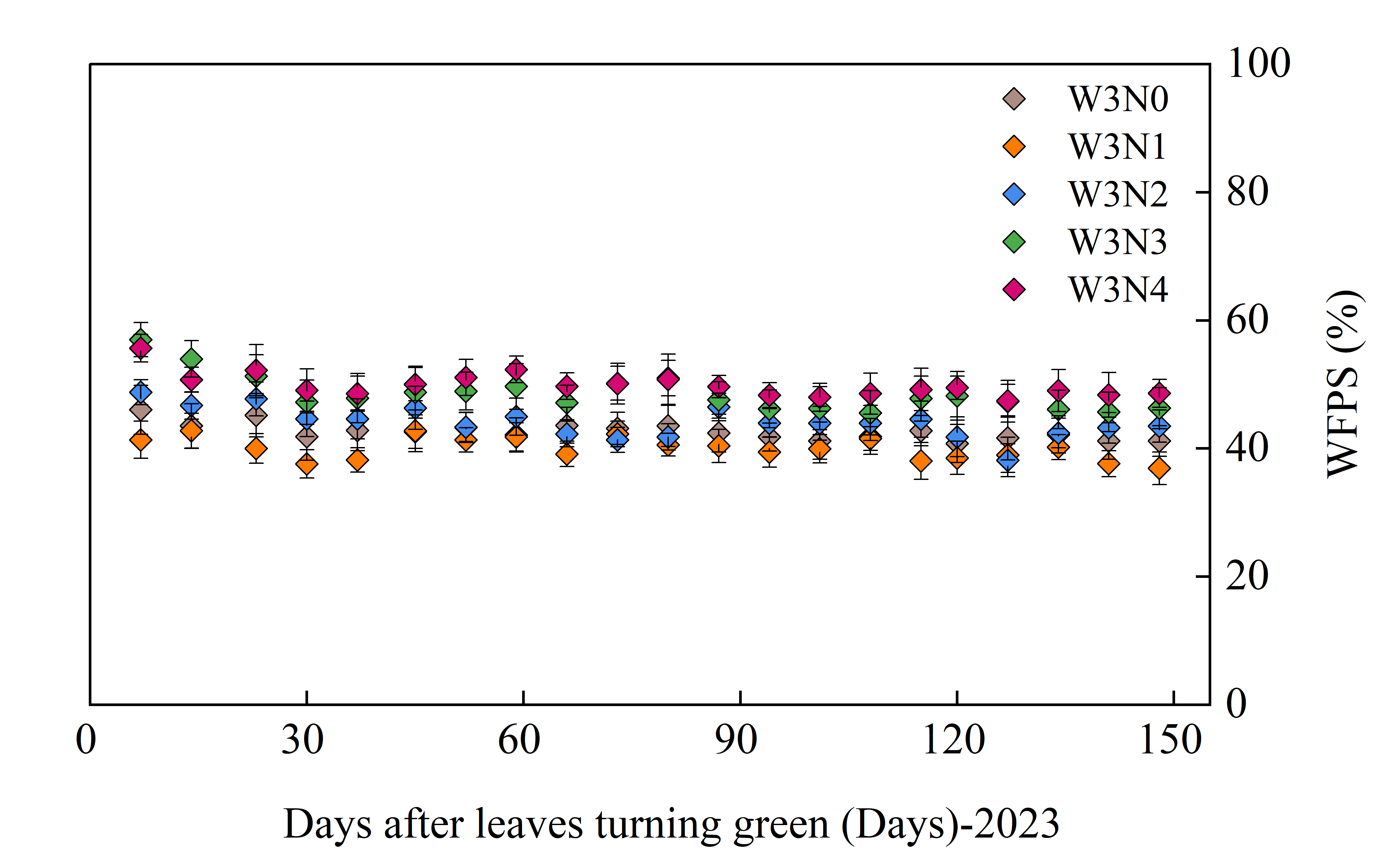


**Figure S2.** Effects of irrigation (W) and nitrogen (N) treatments on the dynamics of soil NH_4_^+^-N content in alfalfa growing seasons in 2022 and 2023. Error bars indicate standard deviation (SD). The red arrow indicates a fertilization event. The processing abbreviations are the same as those described in Figure 2.


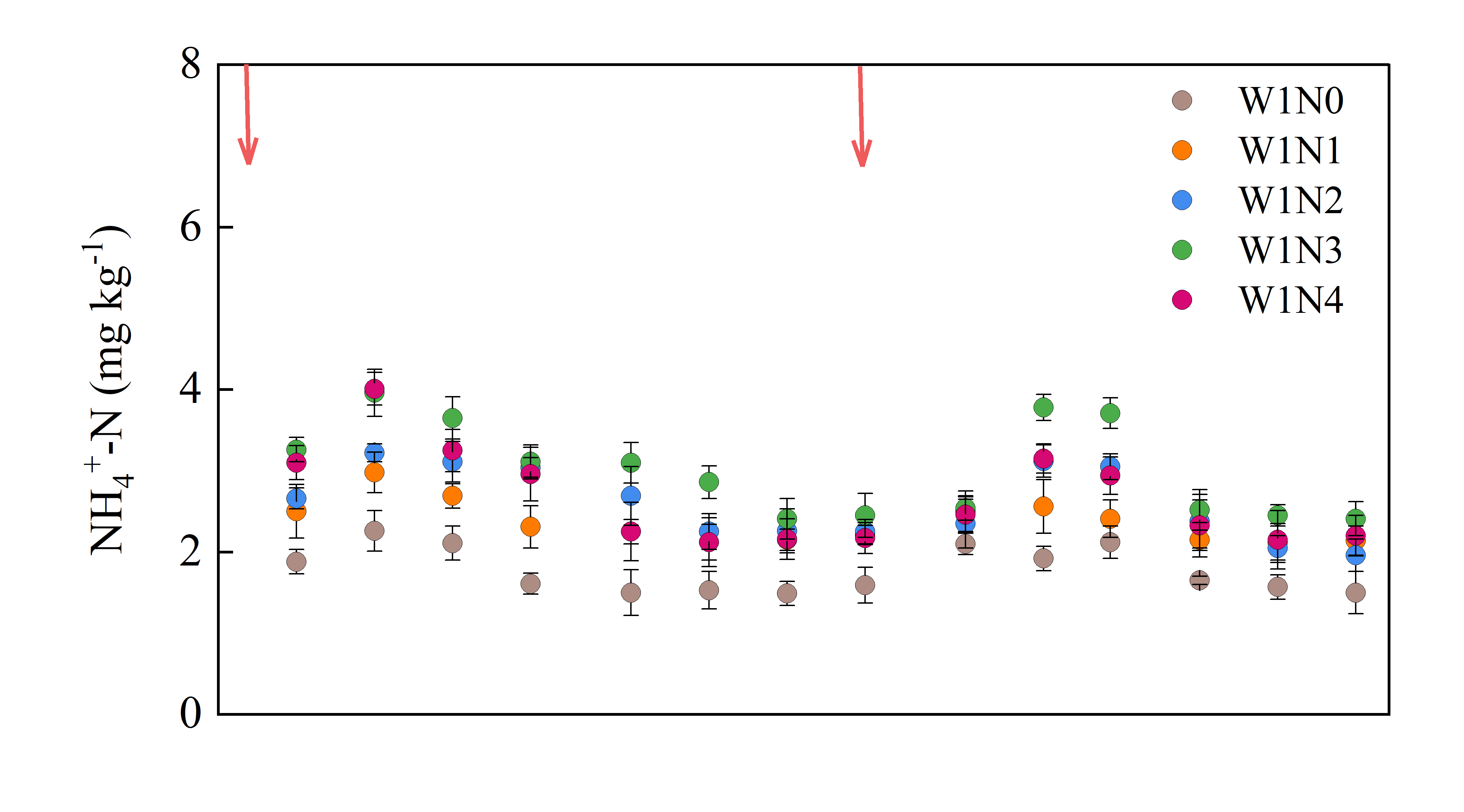

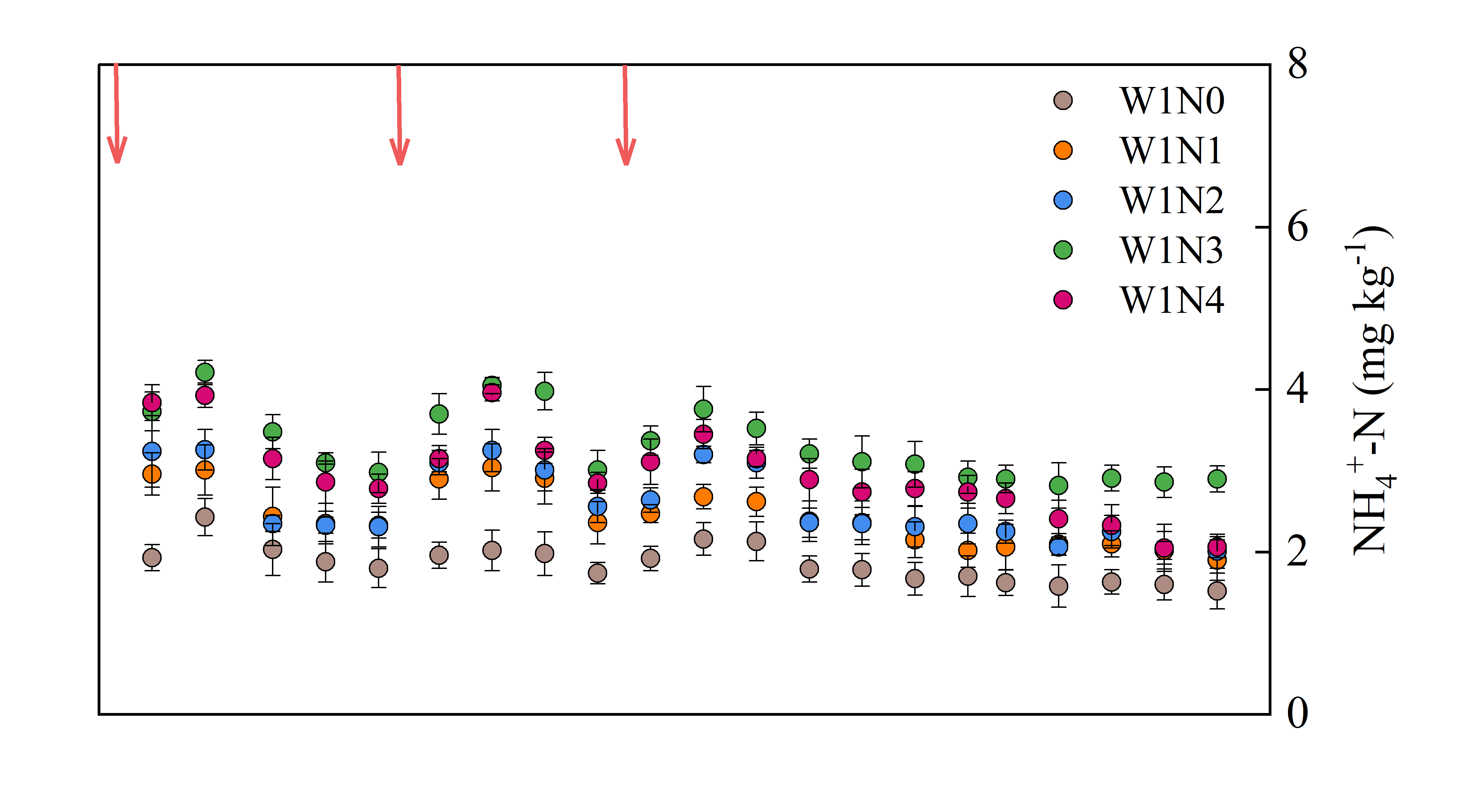

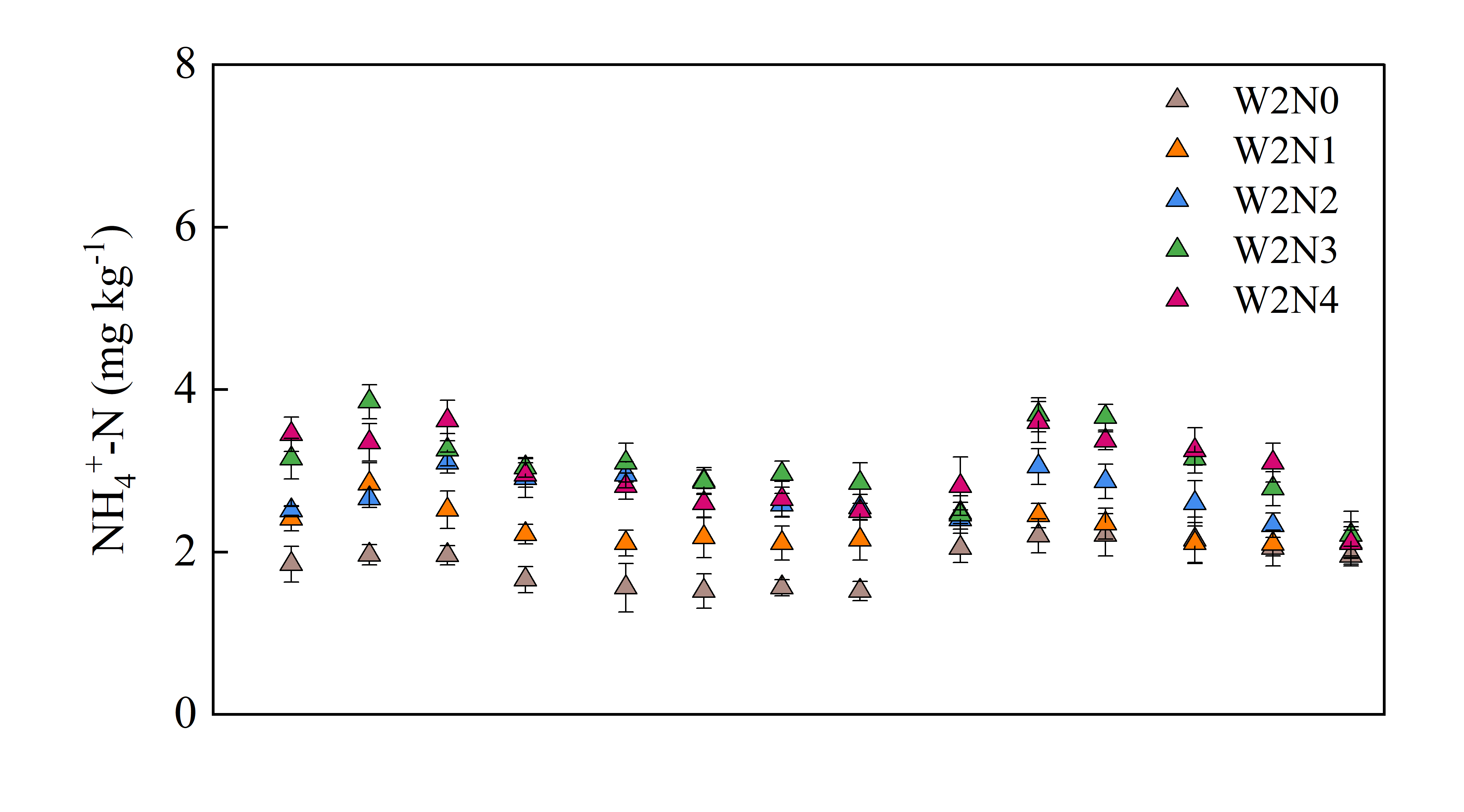

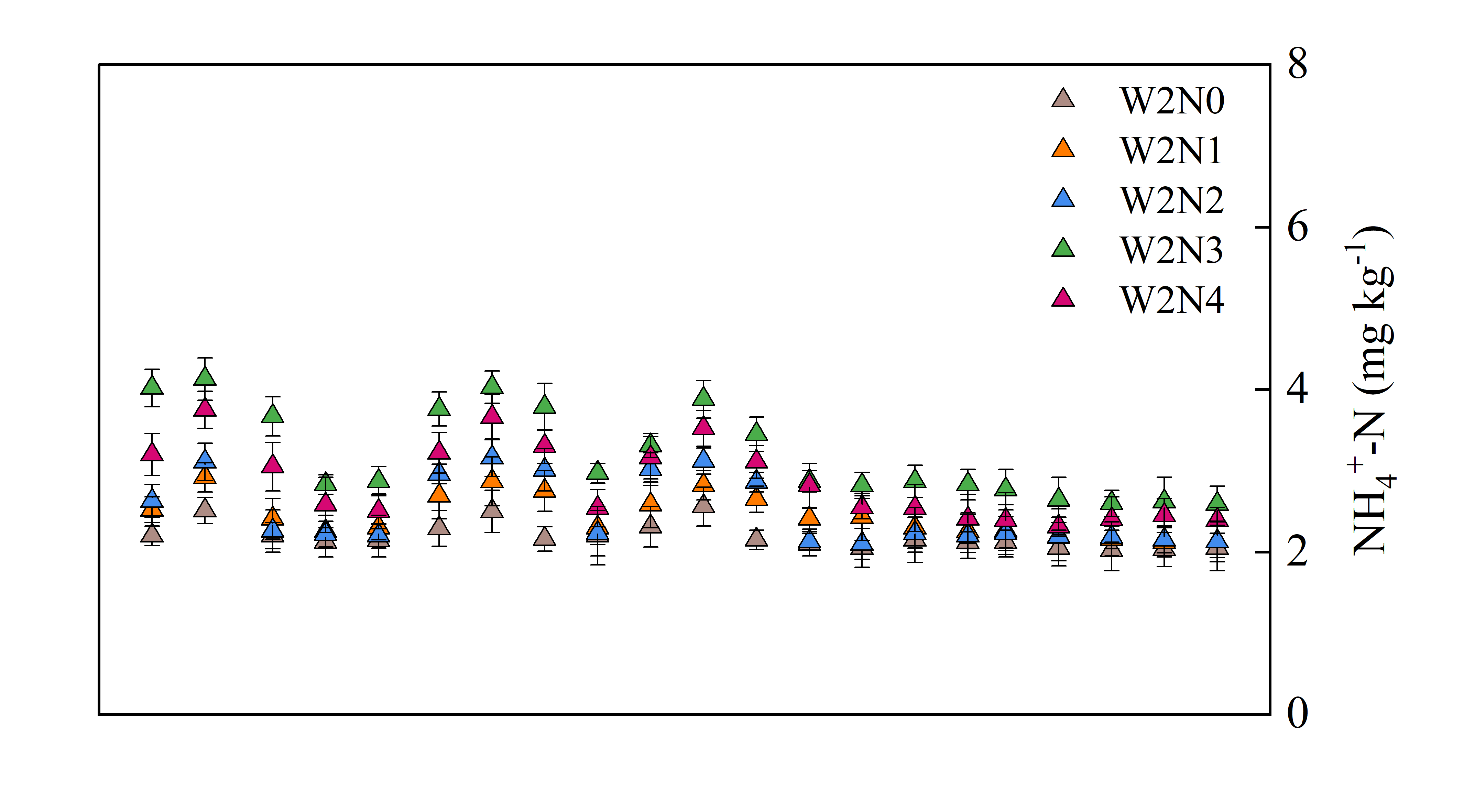

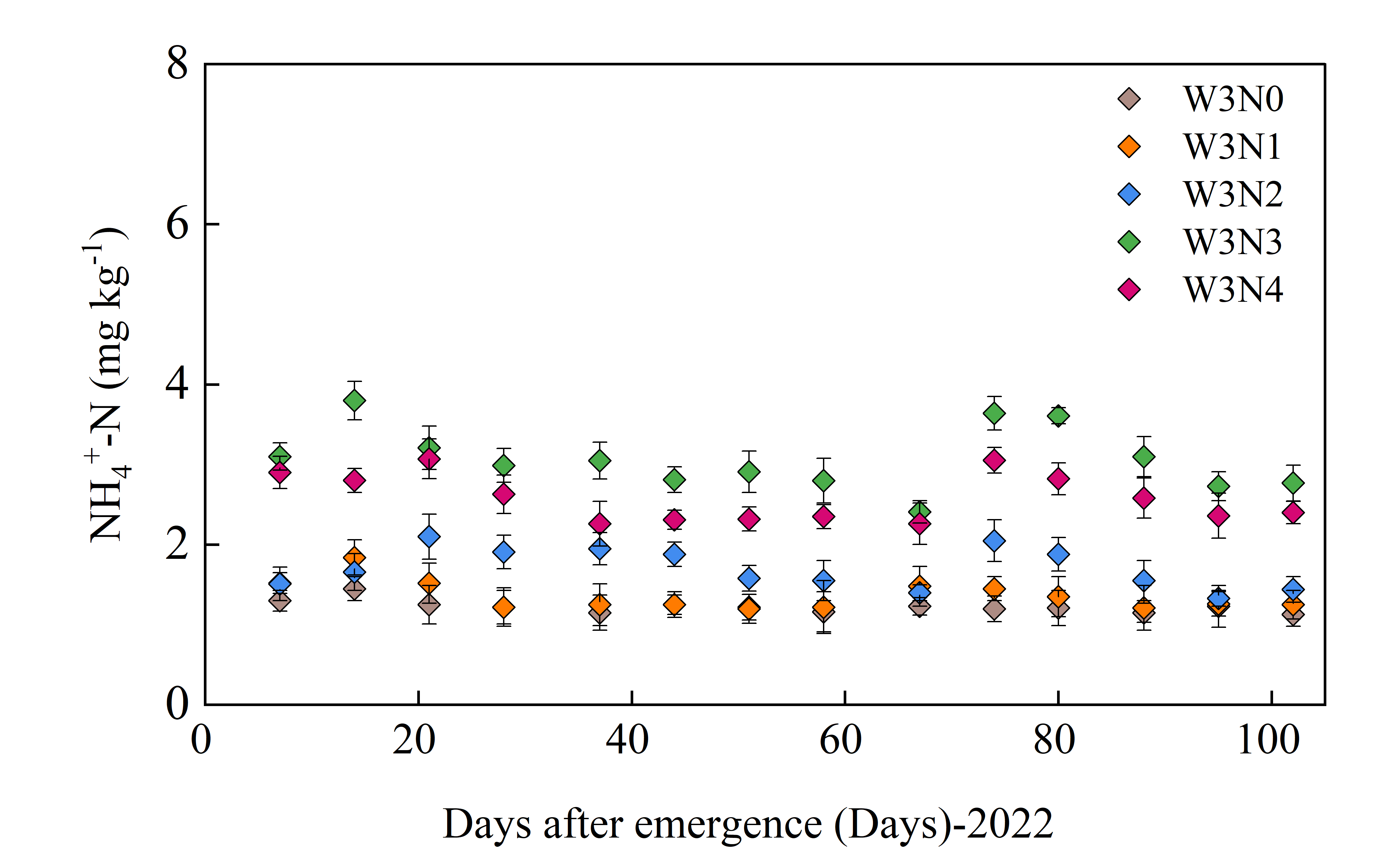

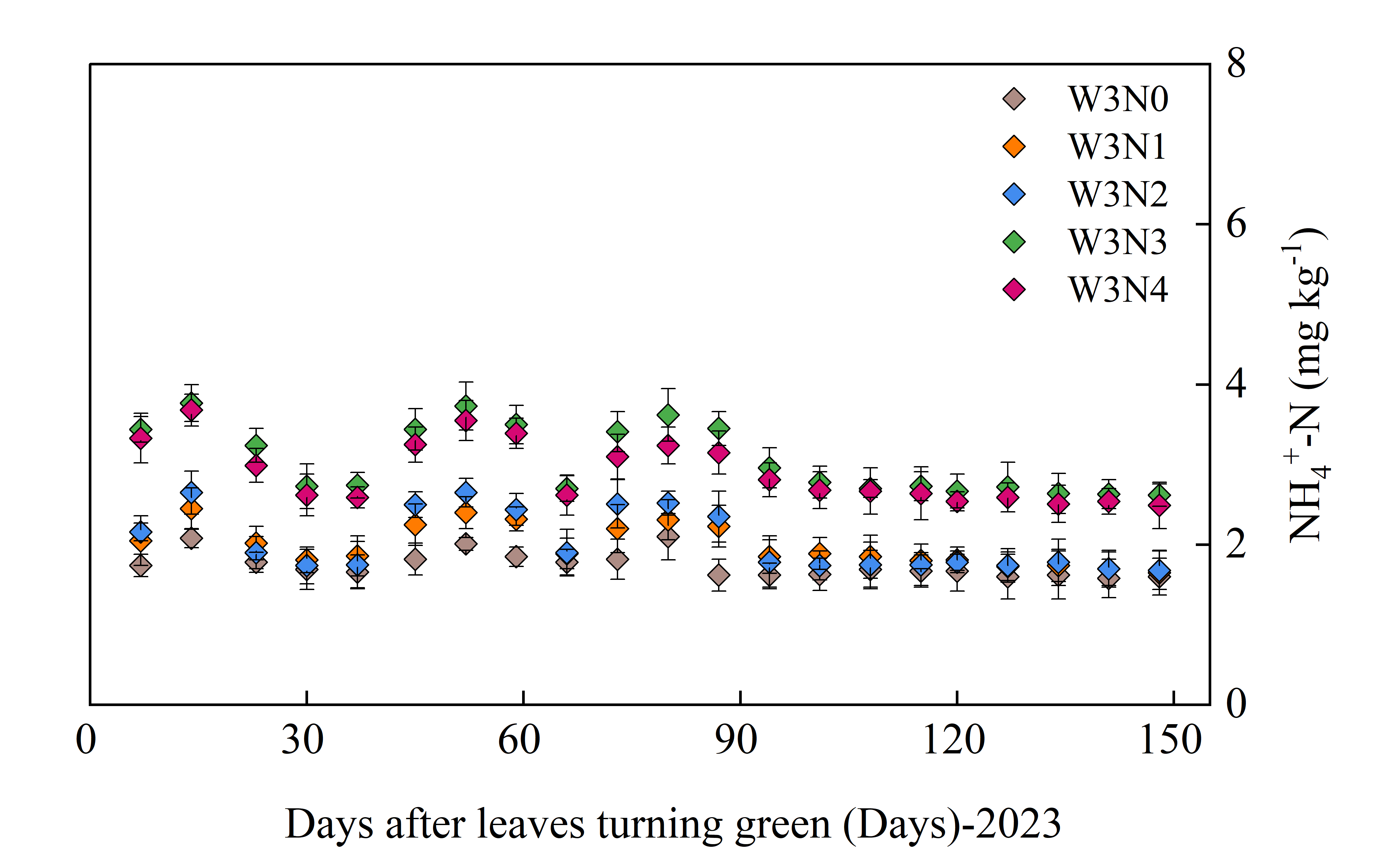


**Figure S3.** Effects of irrigation (W) and nitrogen (N) treatments on the dynamics of soil NO_3_^-^-N content in alfalfa growing seasons in 2022 and 2023. Error bars indicate standard deviation (SD). The red arrow indicates a fertilization event. The processing abbreviations are the same as those described in Figure 2.


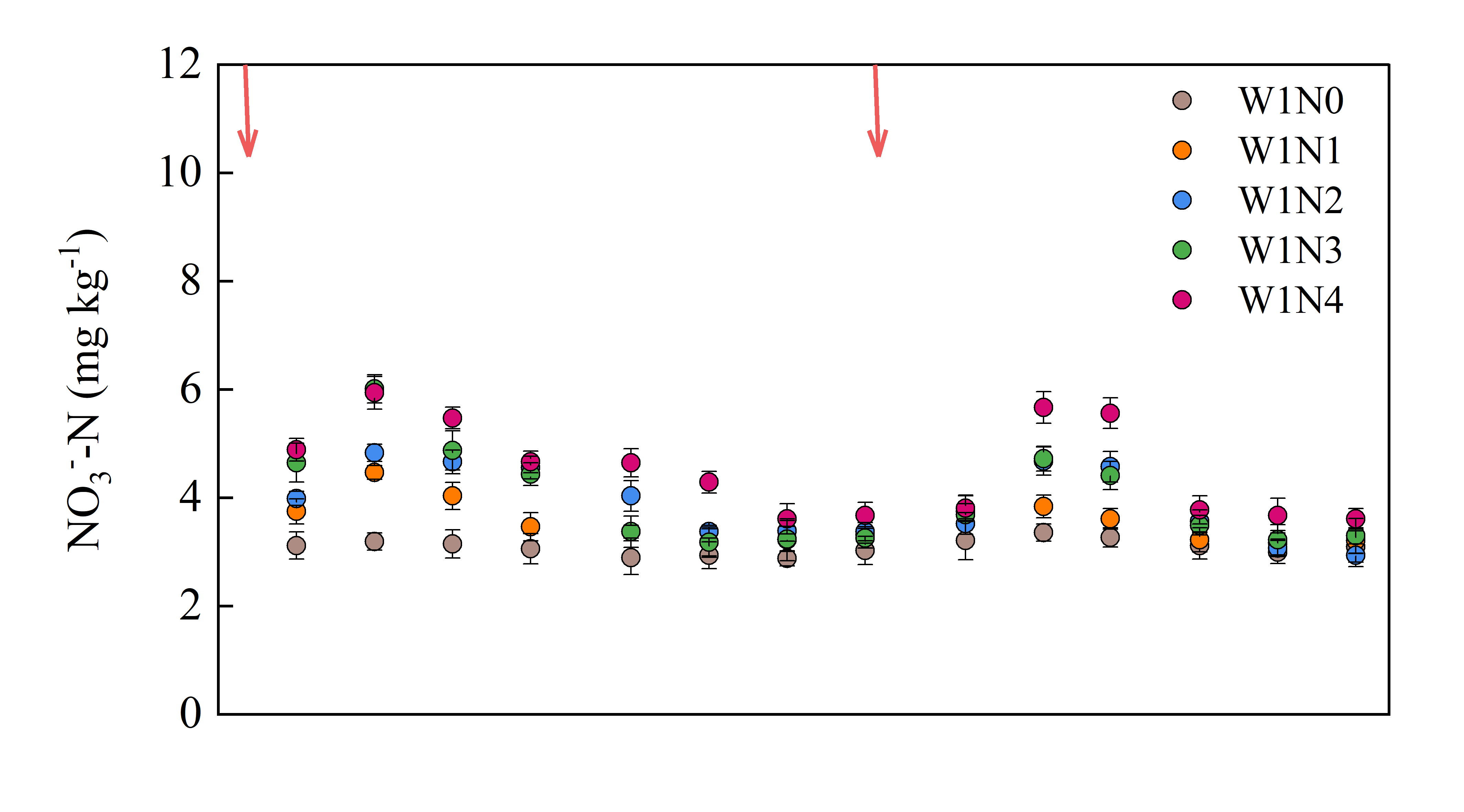

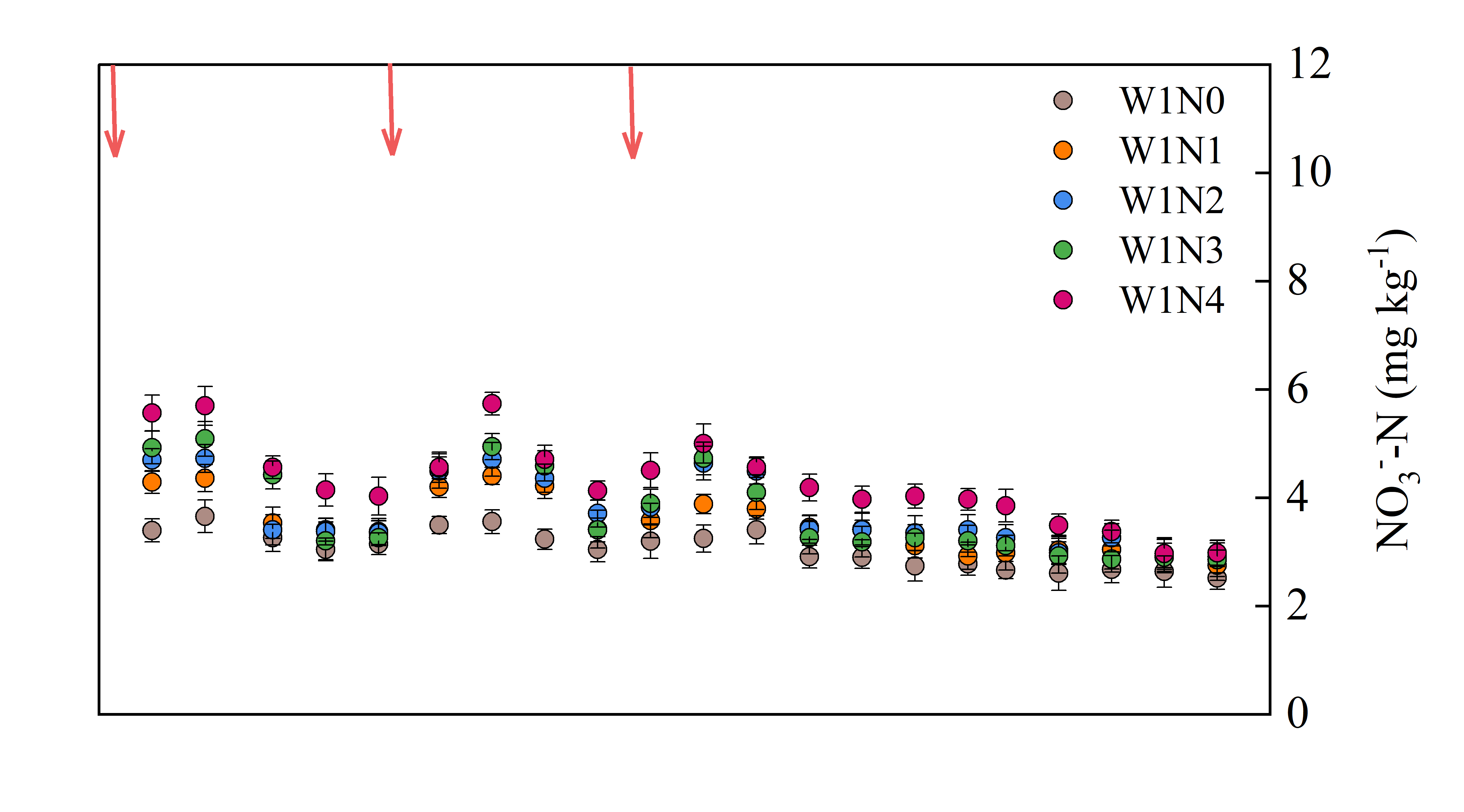

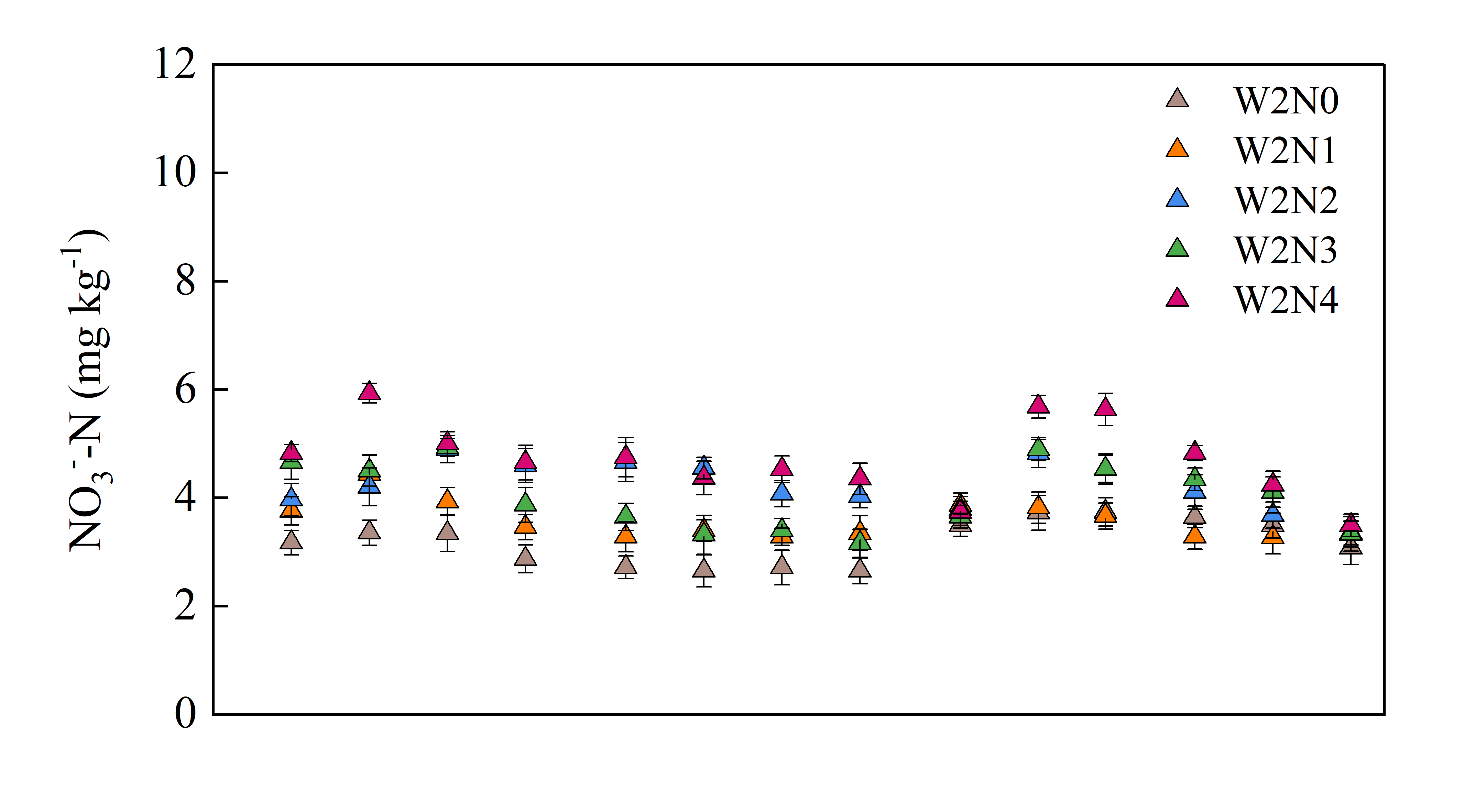

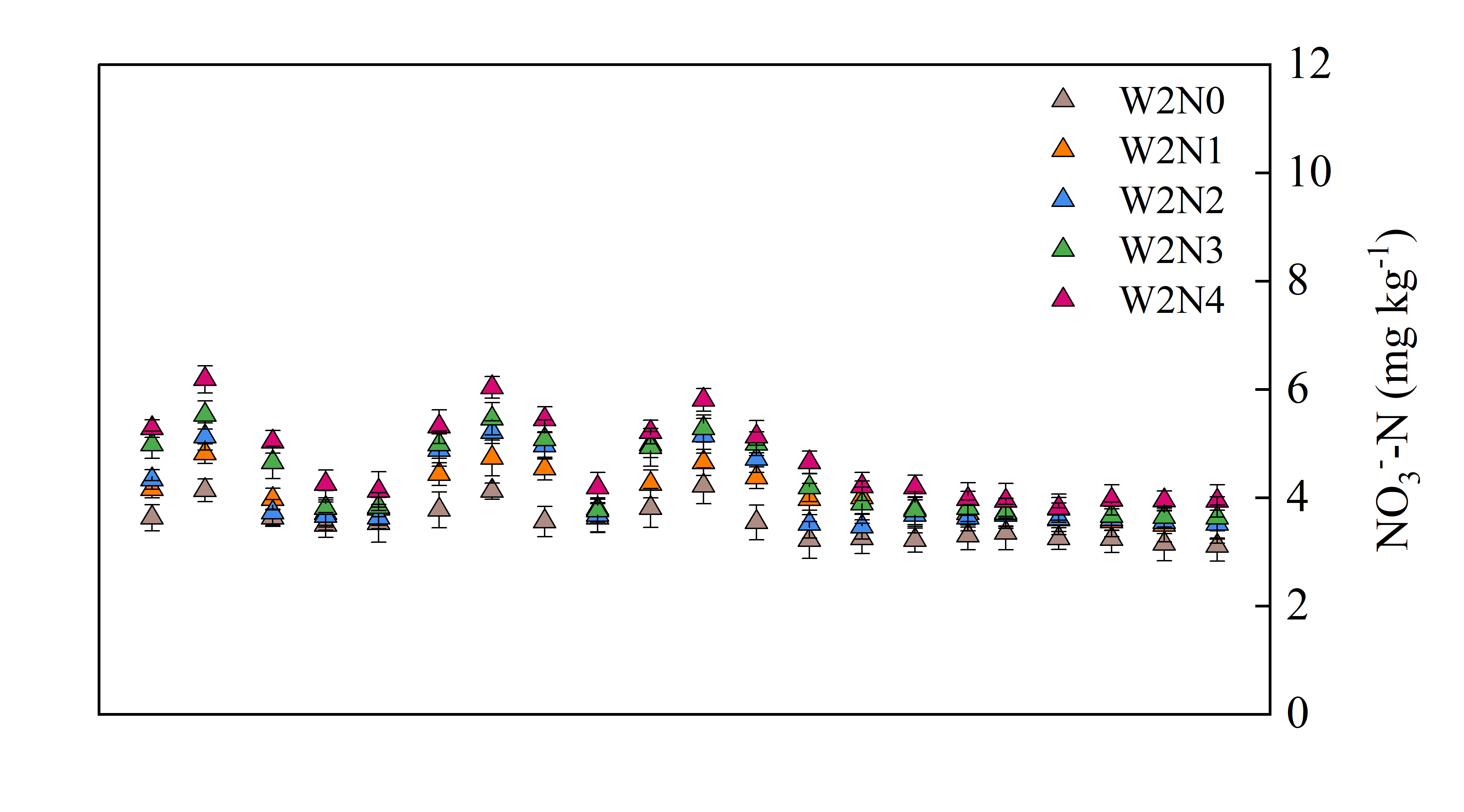

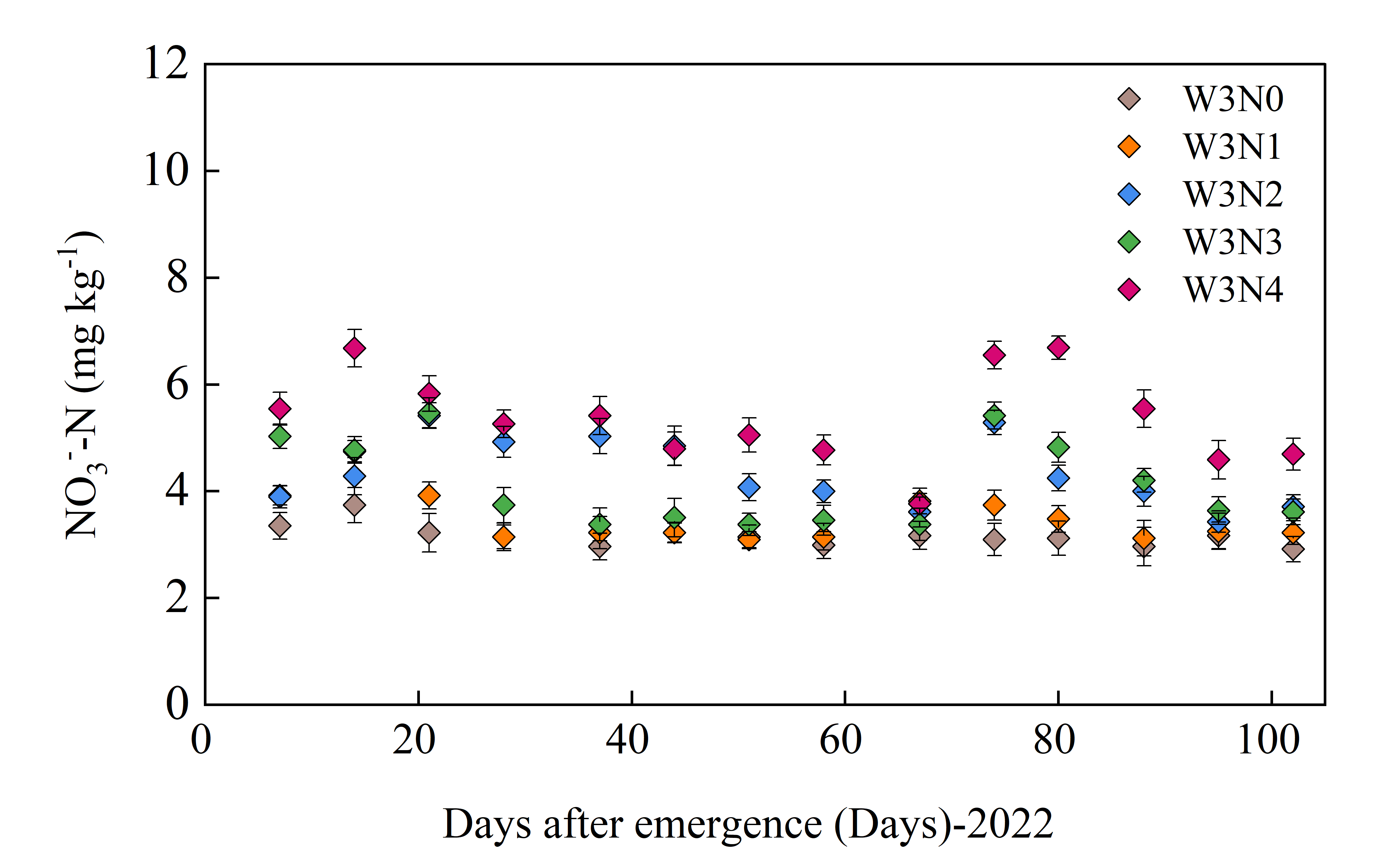

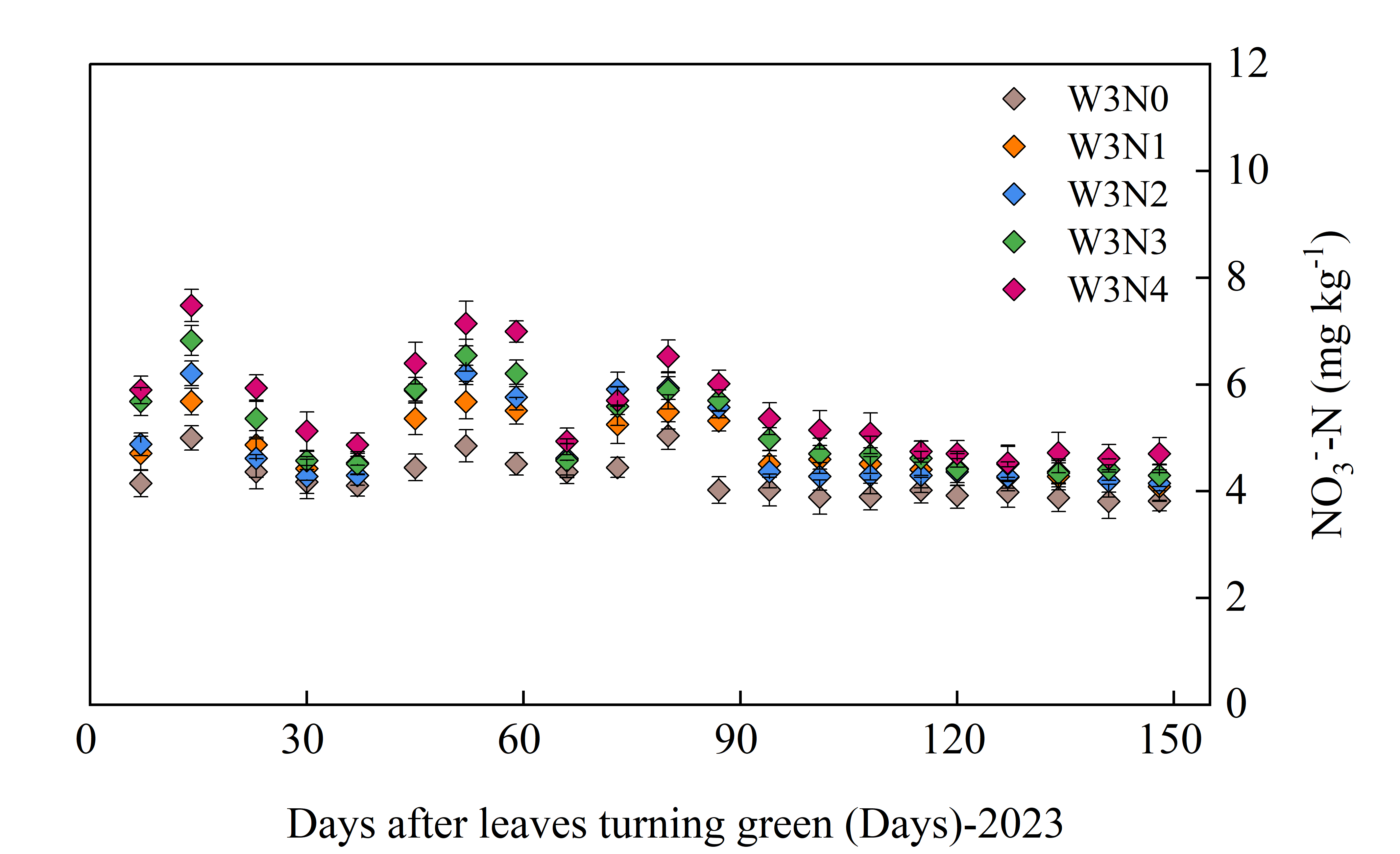


**Figure S4.** Relationship between Water-filled pore space (WFPS), NH_4_^+^-N content, NO_3_^-^-N content, and N_2_O emission flux in alfalfa growing seasons in 2022 and 2023 under different water and nitrogen treatments. **, *p* < 0.01; *, *p* < 0.05.


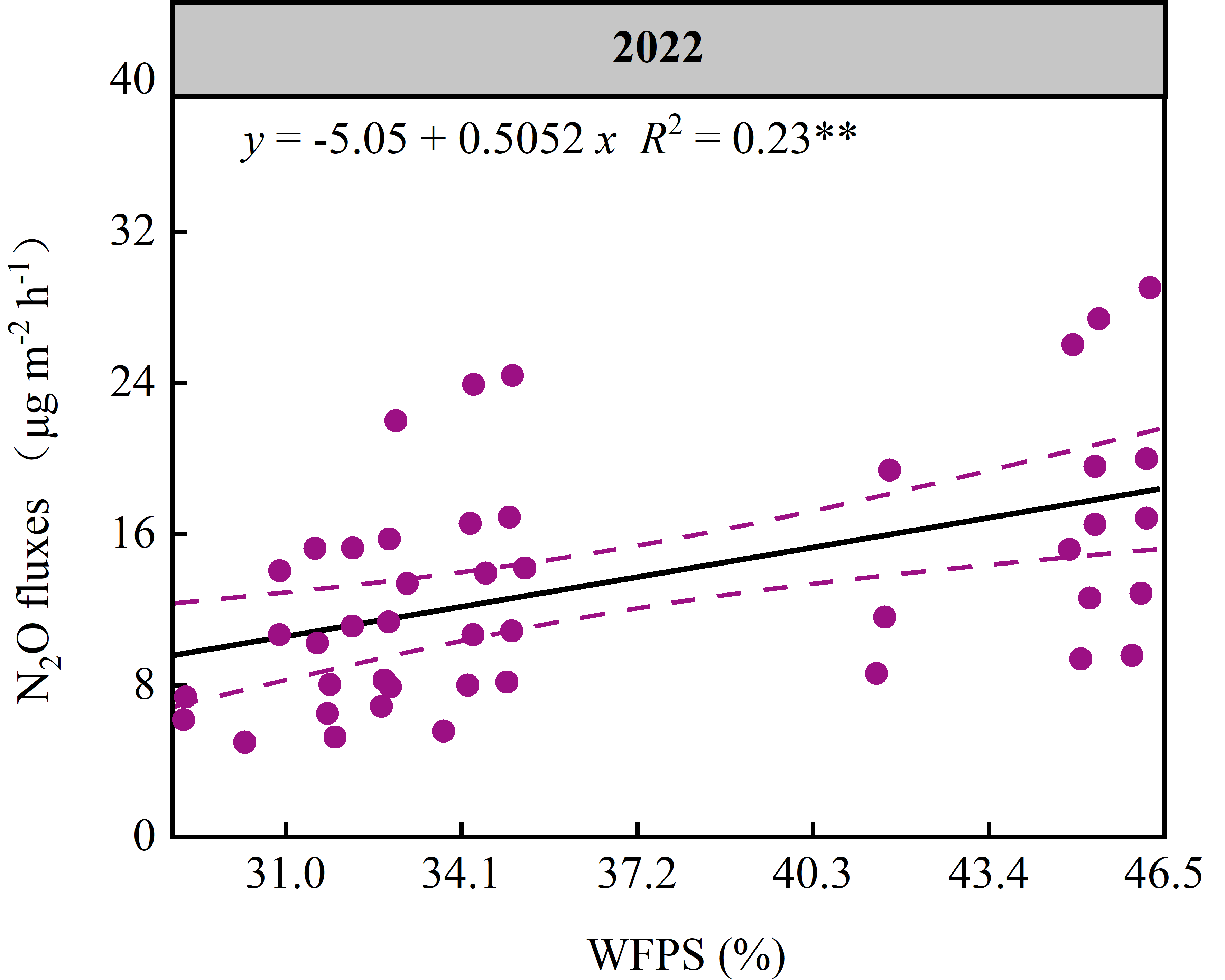

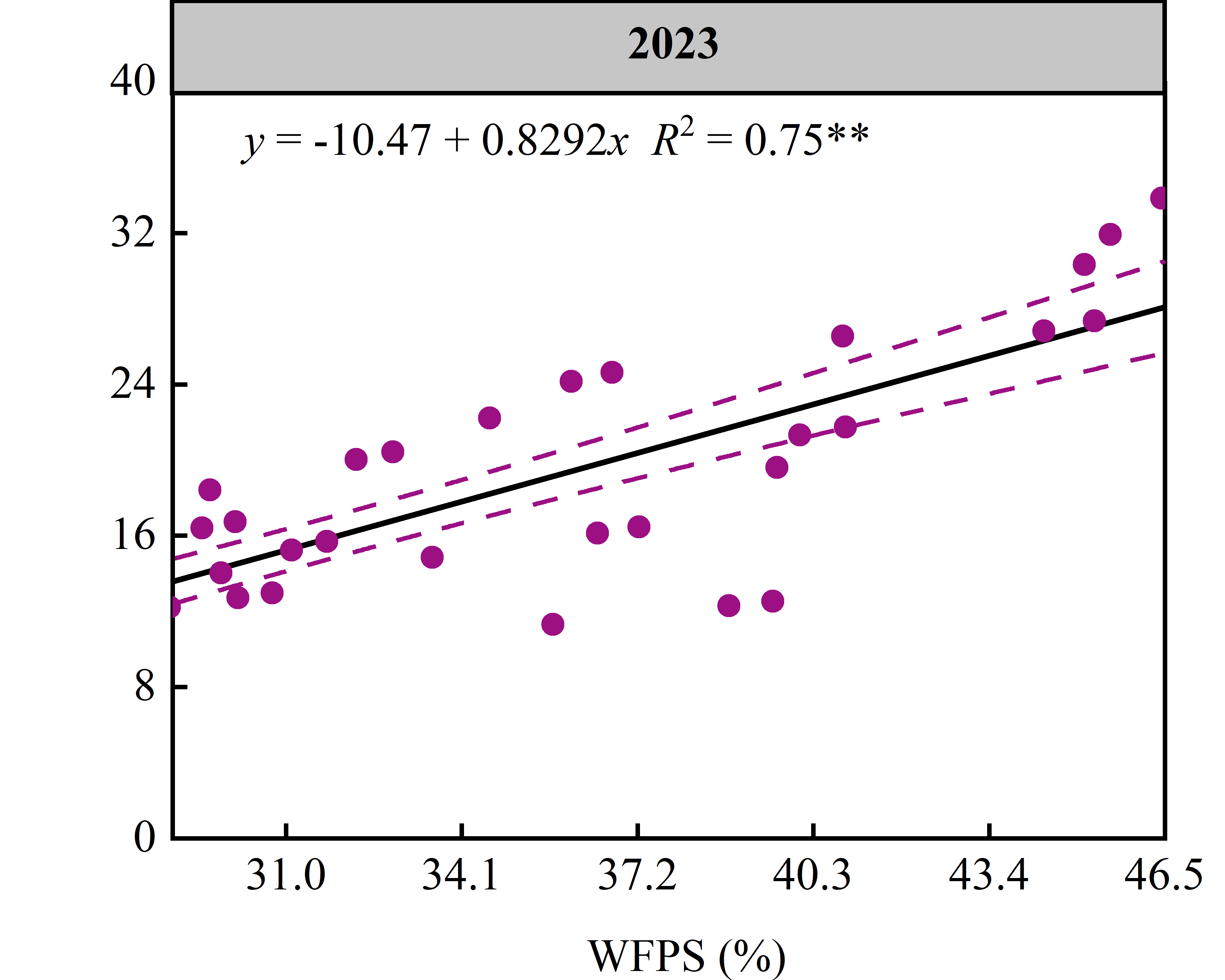

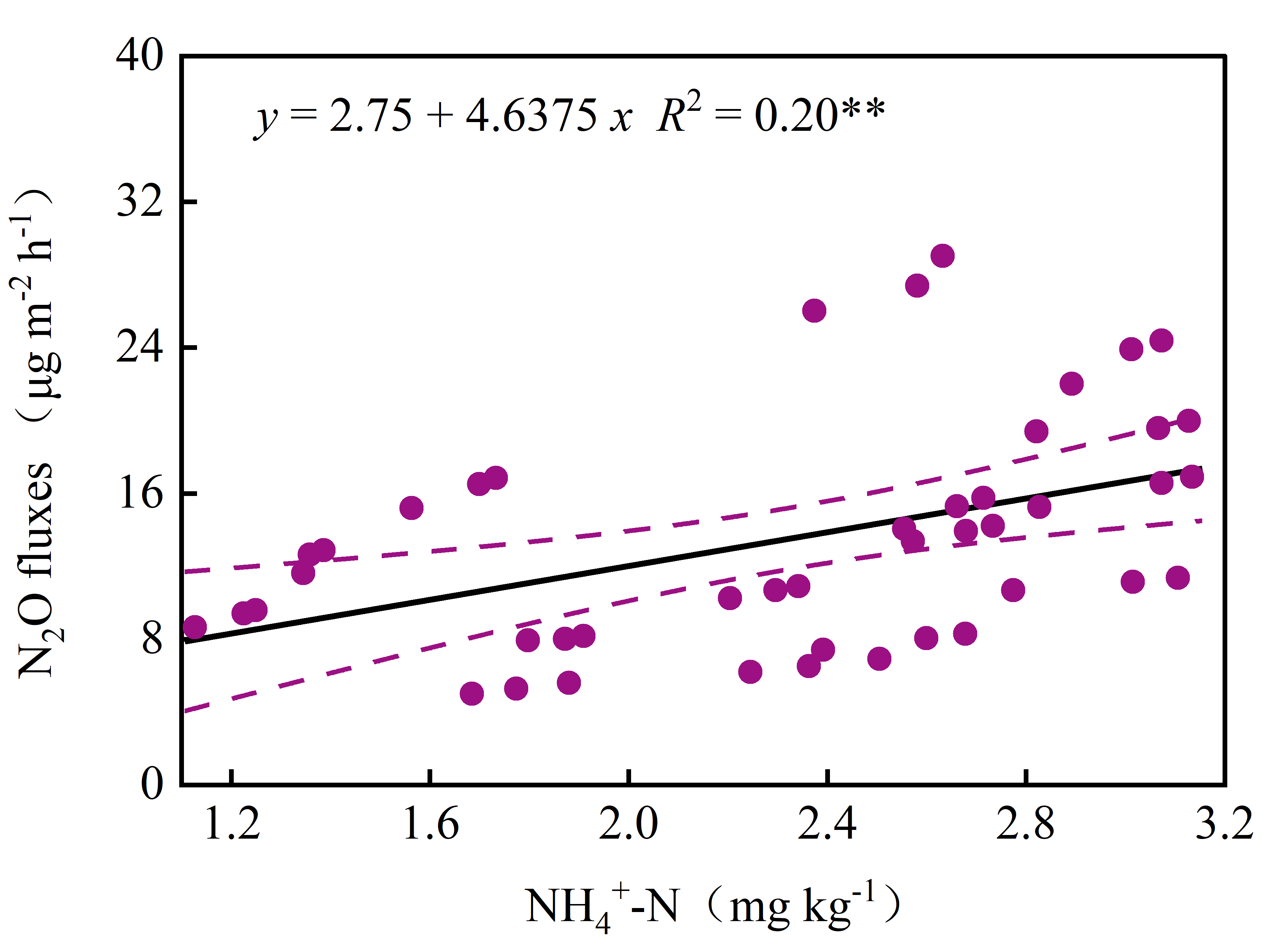

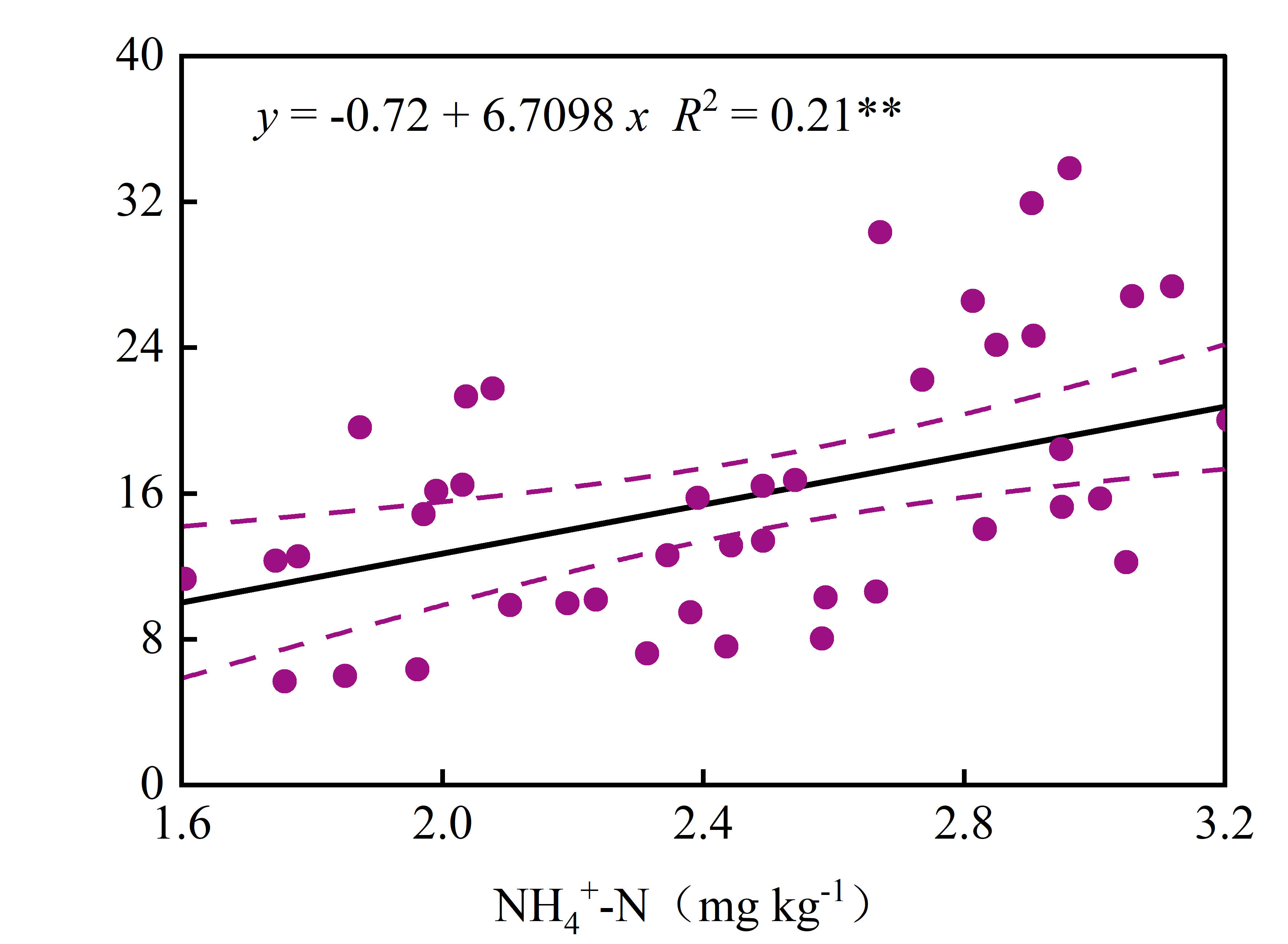

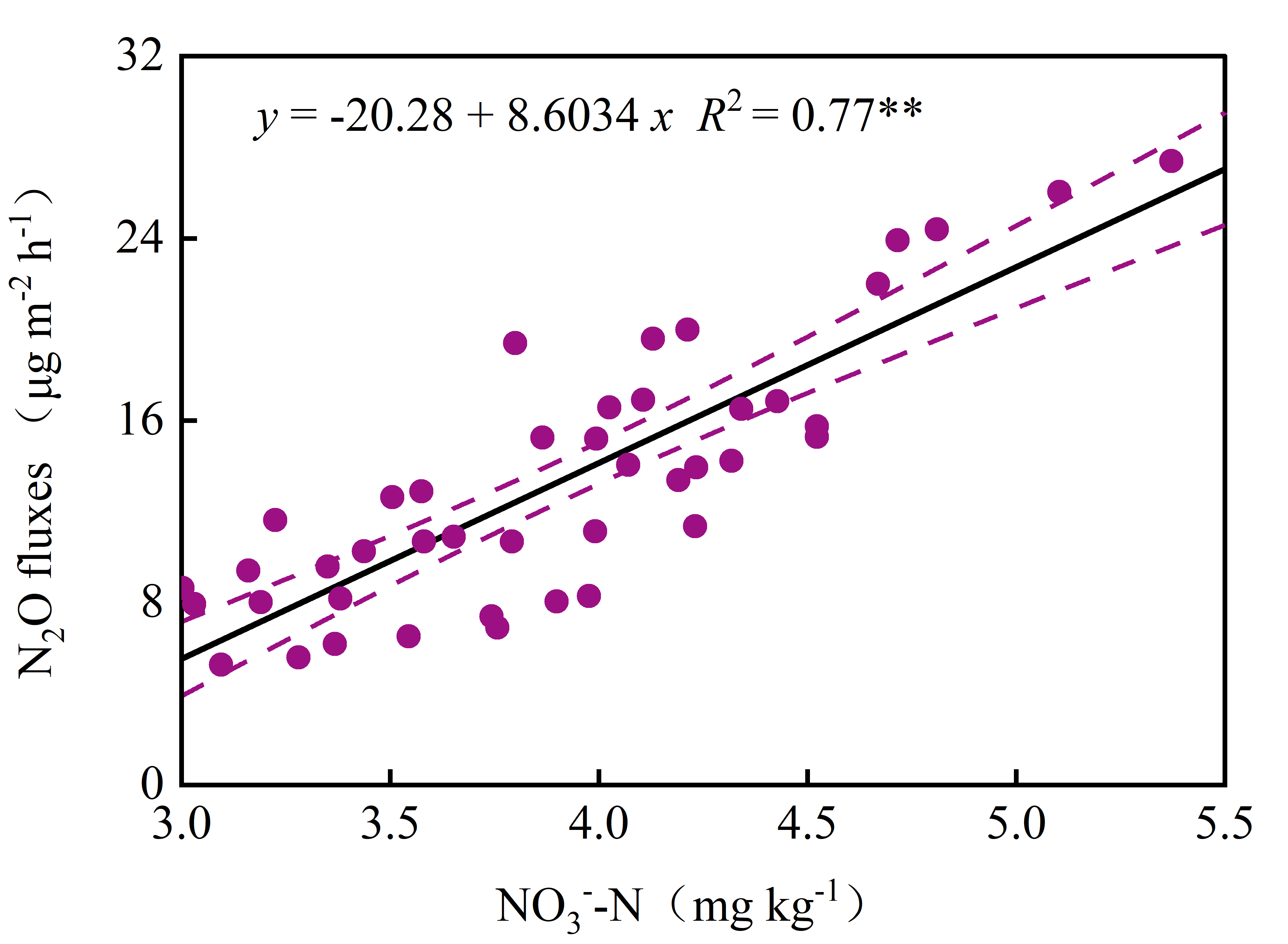

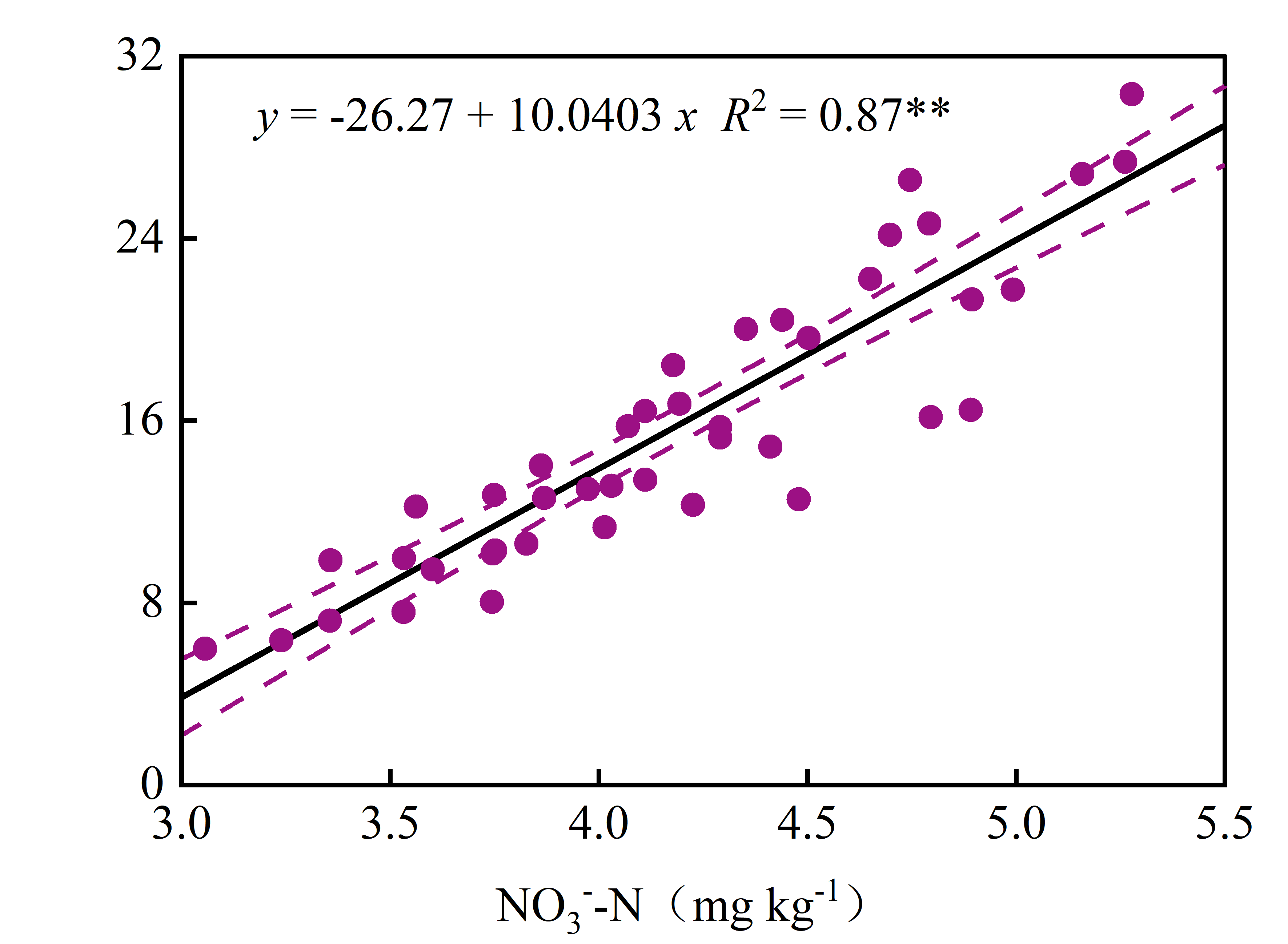

Supplement: Supplementary file 1 [file SupplementaryFile1.docx]
